# Steady-state ^14^CO_2_ tracing reveals early disruption of assimilation–export coupling in tomato under static continuous light but not under dynamic 24-h LED schedules

**DOI:** 10.3389/fpls.2026.1781439

**Published:** 2026-06-09

**Authors:** T.R.J.G. Marie, E.D. Leonardos, N. Rana, M. Pahlevaninezhad, B. Grodzinski

**Affiliations:** 1 Department of Electrical and Computer Engineering, Queen’s University, Kingston, ON, Canada; 2 Department of Plant Agriculture, University of Guelph, Guelph, ON, Canada

**Keywords:** carbon export, circadian rhythm, continuous light, dynamic LEDs, photoperiodic injury, photosynthesis, tomato

## Abstract

Artificial lighting is a major cost in controlled-environment agriculture (CEA), motivating longer photoperiods at lower photosynthetic photon flux density (PPFD) to meet a target daily light integral (DLI). In tomato, static 24-h lighting can induce photoperiodic injury and reduced photosynthetic performance, often discussed as an end-product (triose-phosphate) limitation that constrains inorganic phosphate (Pi) recycling. Dynamic 24-h LED schedules that impose day–night spectral and intensity cues have been proposed to extend photoperiod without injury, but their effects on assimilation–export coupling remain unclear. We investigated whether early dysfunction under static 24-h light reflects a downstream sink-side export bottleneck that limits carbon export regardless of source (newly assimilated and mobilization of stored carbon), or a source-leaf limitation that selectively reduces concurrent export during assimilation while preserving export supported by mobilizing stored carbon, and whether dynamic schedules avoid these constraints. We used steady-state ^14^CO_2_ labeling with open-flow gas exchange to quantify net CO_2_ exchange rate (NCER), concurrent export, ¹^4^C retention, chase-derived reserve-supported export (remobilization), and transpiration in intact source leaves of tomato (*Solanum lycopersicum* “Money Maker”) grown under a 16-h/8-h control, a static 24-h regime, or two DLI-matched dynamic 24-h schedules. After 4 days of static 24-h light (pre-injury), export declined more than NCER, lowering relative export by ~12% and increasing the retained labeled pool. Whole-night remobilization efficiency scaled with retained pool size was conserved across treatments (~30%), including the Day 4 static regime, arguing against an early shared sink/transport bottleneck. After 3 weeks, static continuous light caused severe injury with suppressed NCER, export and reduced water-use efficiency (WUE), whereas dynamic regimes remained injury-free and maintained daytime assimilation–export coupling and WUE comparable to the control. Together, these data indicate that pre-injury static continuous light first disrupts assimilation–concurrent export coupling, a symptom not observed under dynamic 24-h LED schedules.

## 1 Introduction

Artificial lighting enables year-round greenhouse and indoor crop production, but electricity demand associated with lighting remains a major constraint on economic and environmental performance in controlled-environment agriculture (CEA) (Graamans et al., 2018; Harbick and Albright, 2016; Neo et al., 2022). One approach to shift electrical load to off-peak hours and reduce installed lighting capacity is to deliver a given daily light integral (DLI) using longer photoperiods at lower photosynthetic photon flux density (PPFD) (Hao et al., 2018; 2023; Lanoue et al., 2021; Lanoue et al., 2026; Tewolde et al., 2016).Despite these operational advantages, many species exhibit growth plateaus or develop leaf injury when deprived of a sufficiently long dark period, placing a biological ceiling on photoperiod extension (Sysoeva et al., 2010; Velez-Ramirez et al., 2011).

Tomato (*Solanum lycopersicum* L.) is a well-established continuous-light-sensitive crop in both historical and modern controlled-environment studies (Withrow and Withrow, 1949; Hillman, 1956; Velez-Ramirez et al., 2011). Photoperiodic injury in tomato typically presents as mottled/interveinal chlorosis and is frequently accompanied by reduced photosynthetic performance and diminished growth or yield under long photoperiods or continuous light (Demers et al., 1998; Hillman, 1956; Pham et al., 2019). Classic work demonstrated that tomato leaves become chlorotic under photoperiods longer than ~18 h, establishing “photoperiodic chlorosis” as a reproducible phenotype (Withrow and Withrow, 1949). Greenhouse experiments further indicate that photoperiods beyond the optimal range do not reliably improve tomato growth and can even reduce yield (Demers and Gosselin, 2002; Demers et al., 1998). Importantly, responses to continuous light are genotype-dependent, with varying sensitivity across cultivars. Also, a promoter region on a light-harvesting complex gene from wild tomato confers tolerance to continuous light and can enable substantial yield increases when long photoperiods are otherwise limiting (Velez-Ramirez et al., 2014). These observations motivate mechanistic work that identifies which physiological processes fail under continuous light in sensitive tomato and commercial applications of which environmental cues can be used to expand the productive photoperiod window (Velez-Ramirez et al., 2011, 2017).

A physiological explanation for photoperiodic injury is that sustained carbon input can exceed the capacity for end-product commitment (primarily sucrose synthesis and subsequent utilization/export), leading to carbohydrate accumulation, altered partitioning, and feedback downregulation of photosynthesis (Demers and Gosselin, 2002; Dorais et al., 1996). The authors proposed that long photoperiods reduce sucrose-phosphate synthase (SPS) activity in tomato, promoting inorganic phosphate (Pi) limitation, in which reduced Pi availability in the cytosol and/or stroma constrains photosynthetic metabolism and biases carbon toward starch accumulation (Ayari et al., 2000; Dorais et al., 1996). Whether reduced SPS activity primarily reflects feedback from carbohydrate buildup associated with limited sink/transport utilization or a source-leaf limitation in endogenous (time-dependent) SPS activation under weak entrainment cues remains unresolved. Carbohydrate accumulation and impaired photochemistry often co-occur during injury development, and sucrose/starch status has been reported to correlate negatively with PSII maximum quantum efficiency under abnormal light/dark cycles and continuous light in tomato (Velez-Ramirez et al., 2017b).

A key observation is that continuous light does not inevitably cause injury if plants receive a daily temperature cue. Multiple studies show that diurnal temperature fluctuations (thermoperiods) or even a short daily temperature drop can prevent interveinal chlorosis under continuous light and maintain higher photosynthetic performance and PSII function relative to constant temperature under continuous light (Haque et al., 2017; Ikkonen et al., 2015; Shibaeva et al, 2015; Sysoeva et al., 2012). These were interpreted as support for a circadian clock entrained by temperature fluctuations as a protective mechanism (Sysoeva et al., 2012). Also, a diurnal temperature variation (in which the full night period has a lower temperature) under continuous light restored reactive oxygen species (ROS) quenching as a candidate mediator of tolerance (Haque et al., 2017). These outcomes align well with the fact that low-temperature perturbations alter SPS activity through a circadian regulated phosphatase (Jones and Ort, 1997; 1998). This also supports the possibility that photoperiodic injury can emerge when endogenous, time-dependent activation of sucrose-commitment/export capacity is weakened under static 24-h regimes, rather than requiring an initial limitation in reserve mobilization.

Beyond temperature, shifting irradiance and spectrum delivered during the subjective night have also been shown to modulate the severity of photoperiodic injury. In growth chamber studies, diurnal PPFD fluctuations (300 µmol m^−2^ s^−1^ daytime, 150 µmol m^−2^ s^−1^ nighttime) combined with different nighttime spectral treatments found that injury severity was worse under white and blue light quality than under red or orange (Matsuda et al., 2016). However, lowering nighttime white PPFD to 50 µmol m^−2^ s^−1^ (relative to 300 µmol m^−2^ s^−1^ during daytime) displayed very low levels of chlorosis and injury was more correlated with the presence of diel carbohydrate drawdown (turnover) than with absolute pool size (Pham et al., 2019). Greenhouse studies also found an interaction between the amplitude of diurnal variation in PPFD relative to total DLI, where tomato canopies grown under lower DLI had a greater sensitivity to increases in nighttime pure-red (Hao et al., 2025). Lanoue et al. (2026) observed that higher relative blue light in their static continuous white light treatments induced the worst injury, but only under lower DLI during winter, which is consistent with Matsuda et al. (2016) and Hao et al. (2025). However, if pure blue is given at a lower PPFD overnight, there is no injury regardless of the fraction of blue light given during the daytime (Lanoue et al., 2026). Interestingly, static far-red enrichment has also been reported to reduce photoperiodic injury when given throughout the 24-h period, and the effect has been mechanistically linked to phytochrome A (Velez-Ramirez et al., 2019). These studies point to photoreceptor-mediated circadian entrainment as another avenue of photoperiodic injury tolerance.

Together, day–night differences in irradiance, spectrum, and temperature can act as zeitgebers (“time-givers”) that entrain circadian phase to synchronize endogenous coordination among photosynthesis, carbon allocation, stomatal conductance, and antioxidant metabolism (Dodd et al., 2005; Marie et al., 2022). Importantly, carbon metabolism is both a clock output and a clock input; sugars and phosphorylated intermediates show diel rhythms and can feed back on circadian gene expression (Bläsing et al., 2005; Haydon et al., 2013). Thus, the same external cues that modulate injury severity (temperature cycles, day–night PPFD amplitude, and subjective-night spectral composition) may reduce photoperiodic injury in part by restoring coherent circadian timing of source-leaf carbon allocation control, which shapes carbohydrate turnover (including sucrose/starch dynamics) and the propensity to enter Pi-limitation feedback states under extended photoperiods. In this framing, “diel turnover” reflects not only depletion of pools, but restoration of time-of-day regulation of sucrose commitment/export capacity (with SPS activation state as a candidate control point) that supports Pi recycling during the high-flux light period (Bläsing et al., 2005; Haydon et al., 2013; Jones and Ort, 1997; 1998). This framework motivates dynamic 24-h lighting strategies that impose strong entrainment cues and reintroduce diel contrast in carbon balance, with the working hypothesis that improved temporal coordination can lessen downstream metabolic and ROS stress associated with continuous light (Marie et al., 2022).

Despite these advances, key uncertainties remain in how photoperiodic injury develops and, critically, which step in the source–sink continuum first becomes limiting during early continuous-light exposure. Much of the tomato continuous-light literature emphasizes visible injury phenotypes, chlorophyll fluorescence, or endpoint carbohydrate pools measured at a small number of time points (Sysoeva et al., 2010; Velez-Ramirez et al., 2011, 2017). These approaches have been essential for identifying environmental modifiers (temperature cycles, DLI, and spectral cues) and motivating mechanistic hypotheses (circadian asynchrony; Pi/TPU-like feedback; redox/ROS imbalance), but they leave unresolved whether early stress reflects (i) a shared downstream constraint on phloem export (reduced loading/transport and/or sink utilization) that would be expected to depress export of both newly fixed carbon and previously fixed reserves, or (ii) a source-leaf limitation in end-product commitment/export capacity that could selectively reduce assimilation–concurrent export coupling (increasing retention) while leaving reserve-supported export largely proportional. Resolving this distinction matters because mitigation strategies differ if continuous light primarily constrains daytime concurrent export capacity versus imposing a generalized export limitation, and because dynamic 24-h recipes are designed to restore entrainment cues and diel contrast rather than to change total DLI (relative to a conventional DLI target).

A specific gap is that commonly reported carbohydrate pools (leaf sugars and starch) do not uniquely diagnose export limitation. Elevated sugars or starch can result from reduced sink demand, reduced loading/transport, altered sucrose synthesis/Pi limitation, or altered timing of starch turnover; conversely, injury can be alleviated under conditions that increase diel carbohydrate amplitude rather than reducing absolute pool sizes (Pham et al., 2019). Therefore, mechanistic progress requires measurements that directly quantify flux partitioning, how much carbon is (a) fixed, (b) exported concurrently, (c) retained in the source leaf during assimilation, and (d) later exported from previously fixed pools, under the same intact-leaf conditions and time scale relevant to dynamic lighting blocks (Ainsworth and Bush, 2011; Geiger and Fondy, 1979; Grodzinski et al., 1998).

Steady-state ¹^4^CO_2_ labeling coupled to open-flow gas exchange provides a direct way to probe these flux components *in vivo*. By maintaining a constant ¹^4^CO_2_ supply to an intact leaf and continuously monitoring leaf radioactivity while simultaneously measuring CO_2_ exchange and transpiration, this approach estimates concurrent export as net assimilation minus retention once isotopic equilibrium is achieved (after ~1.5 h) (Geiger and Fondy, 1979; Grodzinski et al., 1998; Leonardos and Grodzinski, 2014). When the labeling period is followed by a chase under unlabeled CO_2_, the subsequent loss of label from the leaf quantifies export supported by previously fixed carbon reserves (i.e., mobilization and translocation of retained assimilates), allowing daytime concurrent export to be separated conceptually and quantitatively from post-label export (Leonardos et al., 2003). This distinction is especially important under extended photoperiods because carbon export during the subjective night can reflect a changing mixture of concurrent export supported by ongoing assimilation under dim/continuous illumination and reserve-supported export from previously fixed pools, and the relative contributions of these fluxes cannot be inferred from endpoint pool sizes alone (Stitt and Zeeman, 2012). Importantly, steady-state ^14^CO_2_ does not include remobilization of carbon pools that were formed prior to labeling (multi-day).

In tomato, steady-state ¹^4^CO_2_ labeling has been used to resolve diurnal patterns of photoassimilate translocation and transpiration under different light qualities and intensities, demonstrating sensitivity to lighting variables directly relevant to LED recipe design (Lanoue et al., 2018). Building on this methodological foundation, the present study applies steady-state ¹^4^CO_2_ labeling to compare a conventional 16-h photoperiod control, a static 24-h continuous-light regime, and two dynamic 24-h LED schedules that impose discrete spectral and PPFD cues while maintaining comparable DLI. Our overarching objective was to determine whether dynamic 24-h lighting maintains coupling between assimilation and concurrent export over acclimation, and to use post-label chase measurements to test whether early continuous-light stress reflects a shared export constraint (expected to depress both concurrent and reserve-supported export) versus a selective disruption of assimilation–concurrent export coupling (increased retention) with largely preserved reserve-supported export.

## 2 Materials and methods

### 2.1 Plant material and growth conditions

The protocol used for growth was similar to Marie et al. (2024) with a few modifications. Tomato (cv. “Money Maker”) sown at the University of Guelph (Ontario, Canada) and germinated on wet paper filters in sealed Petri dishes were placed in a plant growth chamber (GC-20 Bigfoot series, Biochambers Inc., Winnipeg, Canada) with environmental conditions set to 25°C (day/night) and LED lighting set to 150 µmol m^−2^ s^−1^ PPFD for the 16-h light/8-h dark photoperiod. After germination (approximately 10 days), seedlings were planted in 4 × 4 × 6 cm cells in germination media (Sungro professional germination mix, Soba Beach, AB, Canada) under a humidity dome. The humidity dome was removed 3–4 days later, and established seedlings were transplanted in 10 × 10 × 12 cm pots in standard growing media (Sungro professional growing mix #1, Soba Beach, AB, Canada) and transferred into another plant growth chamber (GC-20 Bigfoot series, Biochambers Inc., Winnipeg, Canada) for 7 days set to 21°C (day/night), 65% relative humidity, and 300 µmol m^−2^ s^−1^ PPFD for the 16-h light/8-h dark photoperiod (Control, see below lighting treatments). Fertigation was supplied as needed with 20-8–20 fertilizer (Plant Products Inc., Leamington, ON, Canada) mixed in regular tap water [Guelph, Ontario tap water is relatively high in carbonates, pH approximately 7, electrical conductivity (EC) approximately 0.85 mS/cm] and adjusted to a pH of 5.6 with phosphoric acid to a final EC of 1.75 mS/cm. Leaf gas-exchange and ^14^C measurements were conducted 42–60 days after sowing (DAS) on the third or fourth true leaf for all treatments and acclimation stages. In this age range, under our conditions and for the cultivar “Money Maker”, the first inflorescence was nearly visible (~42 DAS) to having the first flower on the inflorescence open (~60 DAS), making all the plants in a similar dominantly vegetative developmental stage. The variability in plant age/development was randomly distributed in all treatments and acclimation stages. Each diurnal measurement was taken on a different biological replicate (no repeated measurements across acclimation stages).

### 2.2 Acclimation treatments

Three separate experiments with varying duration of exposure under the lighting treatments (see below) were performed. Those were 1 day of exposure (Day 1), 4 days of exposure (Day 4), and 3 weeks of exposure (Week 3) experiments. For Day 1 exposure experiments, plants remained in the growth chamber under the Control treatment until they were 6–8 weeks old. Then, they were moved in to our ^14^C-System, a plant growth chamber (GS20 BDAF LT Bigfoot series, Biochambers Inc., Winnipeg, Canada) equipped with a University of Guelph custom-made gas exchange and ^14^CO_2_ labeling system, for 24-h leaf gas exchange and C-export measurements (see below). For Day 4 exposure experiments, plants also remained in the growth chamber under the Control treatment until they were 6–8 weeks old, but 3 days prior to leaf gas exchange and C-export measurements, plants were moved into specific treatment growth chambers (GC-20 Bigfoot series, Biochambers Inc., Winnipeg, Canada) under different lighting treatment. All these treatment chambers had identical environmental conditions (previously calibrated with external sensors) except for the lighting treatments described below. On the fourth day, plants were moved to the ^14^C-System for 24-h leaf gas exchange and C export measurements. For Week 3 exposure experiments, plants remained in Control conditions for 1 week after transplanting and then were placed into their respective treatment chambers until they were 6–8 weeks old. After at least 3 weeks of exposure under the different treatments, plants were moved to the ^14^C-System for 24-h leaf gas exchange and C-export measurements.

### 2.3 Lighting treatments

**Figure 1:**
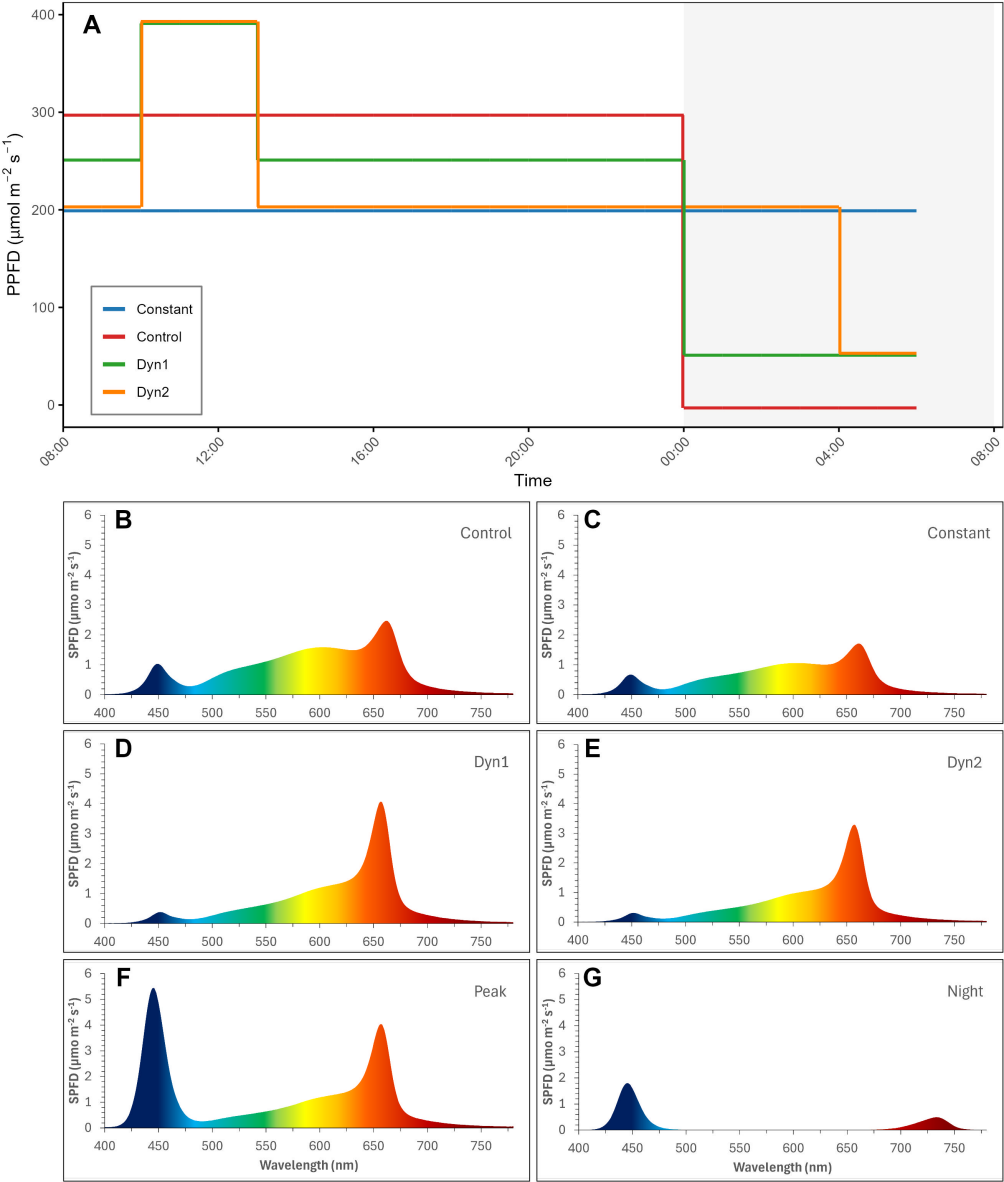
Schedule of light treatments. A single diurnal cycle is represented by non-shaded and shaded background **(A)**. Light intensity is plotted across time of day (08:00 to 08:00 the following day, or 0–24 h of the photoperiod) for each light treatment and sum to equivalent DLIs (17.4 mol m^−2^ day^−1^). Each phase of light quality is labeled with letters denoting a representative spectrum plotted in spectral photon flux density (SPFD) (µmol m^2^ s^1^). Control (red line) and Constant (blue line), 300 and 200 µmol m^−2^ s^−1^ PPFD, respectively, had the same cool-white supplemented with red and far-red spectra **(B, C)**. Dynamic 1 (green line) and Dynamic 2 (orange line) had a “day” spectrum of warm-white plus red for the first 2 h after subjective dawn at 250 and 200 µmol m^−2^ s^−1^ PPFD, respectively **(D, E)**. They then received a “peak” spectrum **(F)** with supplemented pure blue on top of the “day” spectrum to a total of 390 µmol m^−2^ s^−1^ PPFD for 3 h. After the peak phase was finished, they returned to “day” spectrum until the end of their 16- and 20-h photoperiods, respectively. During subjective night, they each received dim blue and far-red, 50 PPFD and 20 µmol m^−2^ s^−1^ PFD, respectively **(G)**. Intensities of spectra of each light treatment are shown in **Table 1**.

Lighting treatments were nearly identical to those of Marie et al. (2024). Each of the four GC-20 Biochambers contained four independently addressable light banks fitted with ballast-compatible T5 replacement LED tubes: red (SKU F54T5HO-LED36R, Growlights Canada Inc., Beamsville, ON, Canada), blue (SKU F54T5HO-LED36B, Growlights Canada Inc., Beamsville, ON, Canada), 3,500-K white (LED25WT5HO/46/835-G8DR, Lumenco Inc., Trois-Rivières, QC, Canada), and 5,000-K white (LED25WT5HO/46/850-G8DR, Lumenco Inc., Trois-Rivières, QC, Canada). Depending on the assigned treatment, designated banks were scheduled to deliver one of four spectral regimes: Control (fixed, unchanging spectrum with a 16-h photoperiod), Constant (fixed, unchanging spectrum with a 24-h photoperiod), Dynamic 1, or Dynamic 2 (both varying spectrum and intensity over the course of the day) (**Figure 1**). Spectral transitions were implemented using the Biochamber Control software to time individual banks, with additional far-red provided by separate LED fixtures on an external timer (FGI Far Red, FARREDLB, Forever Green Indoors Inc., Seattle, WA, USA). Total DLI and far-red DLI were matched across all treatments (**Table 1**).

**Table 1:** Daily light integral (DLI, mmol m^-^² day^-^¹) and average hourly photon flux density (PFD, μmol m^-^² s^-^¹) shown for each treatment.

	Control		Constant		Dynamic 1				Dynamic 2			
	“Day”		“Day”		“Day”	“Peak”	“Night”		“Day”	“Peak”	“Night”	
	16 h		24 h		13 h	3 h	8 h		17 h	3 h	4 h	
	PFD	**DLI**	PFD	**DLI**	PFD	PFD	PFD	**DLI**	PFD	PFD	PFD	**DLI**
Blue	39.6	**2.3**	25.7	**2.2**	16.2	156.7	47.8	**3.8**	13.1	156.7	47.8	**3.2**
Green	114.6	**6.6**	76.1	**6.6**	71.0	70.4	0.4	**4.1**	57.5	70.4	0.4	**4.3**
Red	147.9	**8.5**	99.7	**8.6**	164.4	164.4	1.8	**9.5**	131.6	164.4	1.8	**9.9**
Far-Red	15	**0.9**	10	**0.9**	7.1	10.3	17.4	**0.9**	5.4	10.3	17.4	**0.9**
PAR	300.0	**17.4**	200.0	**17.4**	249.0	390.0	50.0	**17.4**	203.2	390.0	50.0	**17.4**

The table corresponds to the spectral/light schedule in **Figure 1**. Values measured in a central position at canopy height in growth chambers and inside the leaf chamber.

The ^14^C-System plant chamber was fitted with two different LED light fixtures. In one-half of the chamber, a light fixture with eight T5 LED tubes (five CW, one WW, and two R) (Fusion Bright, Growlights Canada Inc., Beamsville, ON, CAN) was used to provide either the Control or the Constant light treatment. In the other half, a custom dynamically controllable LED fixture (Genoptic LED Inc) with five LED banks (FR, two WW, Bl, and R) was used to provide either Dynamic 1 or Dynamic 2 light treatments. The growth chamber was separated in the middle with a black cloth to isolate different light treatments.

### 2.4 Leaf gas exchange and ^14^C export protocol

Leaf gas exchange and ¹^4^C export were measured using an open-flow infrared gas analysis (IRGA) and steady-state ¹^4^CO_2_ labeling system. Full methodological details, background, and calculations are provided in the **Supplementary Material**. Briefly, whole plants were held in a growth chamber and a mature leaflet was enclosed in a temperature-regulated leaf chamber before the start of the photoperiod. Four leaf chambers were run in parallel with a reference line. CO_2_ and H_2_O concentrations in the inlet and outlet air streams were measured sequentially with an IRGA, and NCER, transpiration, and stomatal conductance were calculated from gas concentrations, flow rates, leaf and air temperature in the chambers, and enclosed leaf area.

Labeled CO_2_ was generated from Ba¹^4^CO_3_ and injected into the incoming gas stream to maintain steady-state ¹^4^CO_2_ specific activity, which was monitored using a reference Geiger–Müller (GM) detector and a calibration curve based on trapped inlet samples. In the daytime-labeled experiments, leaves received steady-state ¹^4^CO_2_ for 16 h followed by an 8-h chase without label. In a separate nighttime-labeled protocol, ¹^4^CO_2_ was supplied for 16 h starting in the afternoon to span the photoperiod boundary and the subsequent subjective night. A Geiger–Müller detector permanently positioned beneath each chamber continuously monitored ^14^C retained in the enclosed leaf area. During steady-state ^14^CO_2_ labeling, the instantaneous/concurrent export rate of newly assimilated C was calculated as assimilation rate (NCER from IRGA measurements) minus retention rate (from GM readings corrected for GM efficiency and inlet air ^14^C specific activity). Importantly, it labels only newly assimilated carbon, meaning it underrepresents actual carbon export that remobilizes previously fixed carbon earlier in the day and/or multiple previous days. Relative export was calculated as export divided by assimilation multiplied by 100. During chase periods, export estimates of previously fixed C were calculated based on the disappearance rate of labeled C (from GM readings corrected for GM efficiency and inlet air ^14^C specific activity). Preprocessing of data was performed in Excel to calculate slopes of GM traces and SAS (SAS Institute Inc., 2025) to calculate half-hourly means of NCER, export, relative export, E, gCO_2_, C_i_, and water-use efficiency (WUE).

### 2.5 ^14^C-partitioning

Immediately after each experimental run, leaves were taken out of the leaf chambers in order to determine ^14^C amounts in various fractions (e.g., sugars and starch). The area of the leaf enclosed by the chamber was imaged to determine the leaf area then extracted three times using 80% ethanol at 70°C for 30 min, leaving an ethanol soluble fraction and ethanol insoluble fraction. Ethanol soluble fractions were then dried and suspended in a mixture of water and 99% chloroform (3:2 v/v), agitated, and centrifuged at 11,000 RPM to separate a water soluble fraction (primarily sugars) from chloroform soluble leaf components (pigments, lipids, etc.). Ethanol insoluble fractions (primarily starch) were oven dried at 70 °C for 48 h, dry ground, and suspended in 80% ethanol. The ^14^C content of each fraction was determined using liquid scintillation counting.

### 2.6 Statistical analysis

All statistical analyses and figures were generated in R (v4.5.1) (R Core Team, 2025). Data were analyzed by one- or two-way analysis of variance (ANOVA), as appropriate, with lighting treatment and acclimation state (Day 1 vs. Week 3) treated as fixed effects. Where significant effects were detected, *post-hoc* mean separation was performed using Tukey’s HSD test. Model assumptions were assessed from residual diagnostics. Data are reported as least-squares means ± SE (*n* = 4), with significance accepted at *p* < 0.05.

## 3 Results

### 3.1 Pre-injury short-term continuous light increases carbon retention by disproportionately reducing concurrent export

**Figure 2:**
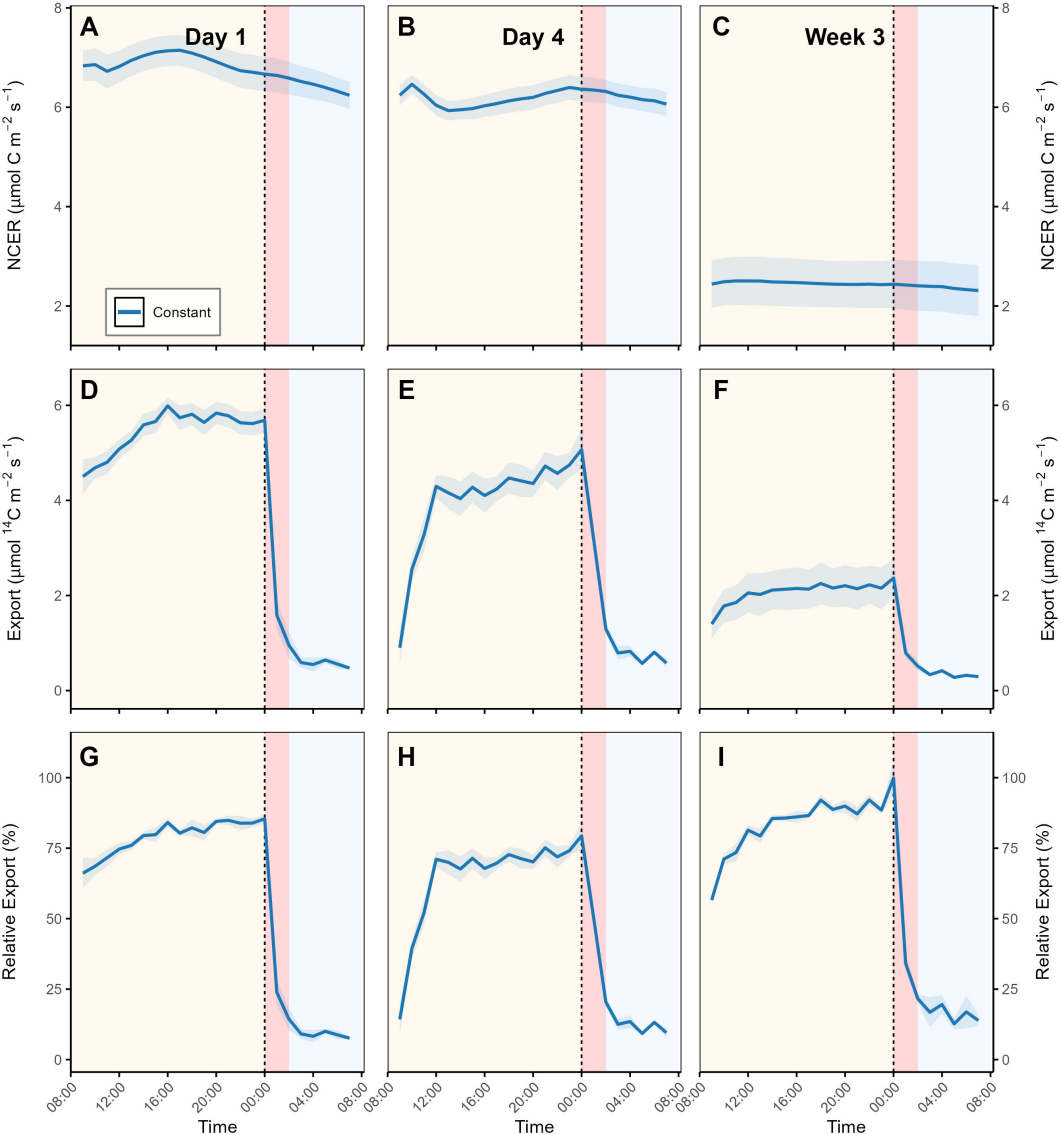
Diel carbon fluxes for the Constant treatment acclimation on Day 1, Day 4, and Week 3. Net CO_2_ exchange rate [NCER; top row, **(A–C)**], ¹^4^C export rate [middle row, **(D–F)**], and relative export [bottom row, **(G–I)**] are shown over a 24-h period under the Constant lighting regime. The *x*-axis is expressed as clock time from 08:00 to 08:00 the following day (0–24 h). Columns correspond to Day 1 (left), Day 4 (middle), and Week 3 (right). Lines are hourly means (*n* = 4) ± SE. Background shading indicates the 16-h ^14^CO_2_ feeding period (light yellow, 0–16 h after lights-on), the initial 2 h nighttime chase window (red, 16–18 h), and the remaining 6-h chase period (light blue, 18–24 h). The vertical dashed line marks the end of the ^14^CO_2_ feed (16 h). Note that, during chase, the calculation of export no longer depends on NCER as there is no concurrent export; instead, it is calculated as the loss of retained labeled C over time. The lack of labeling during the chase results in a drastic drop in export. A vertical dashed line marks the end of the feed at 16 h. NCER and export are expressed in µmol C m^-^² s^-^¹, and relative export is calculated as (export rate/NCER) × 100%. Also, note that relative export during the nighttime chase period uses a different formula that normalizes to each plant’s retained pool at 16 h and is termed remobilization efficiency (**Table 5**;**Supplementary Table 3**).

**Figure 3:**
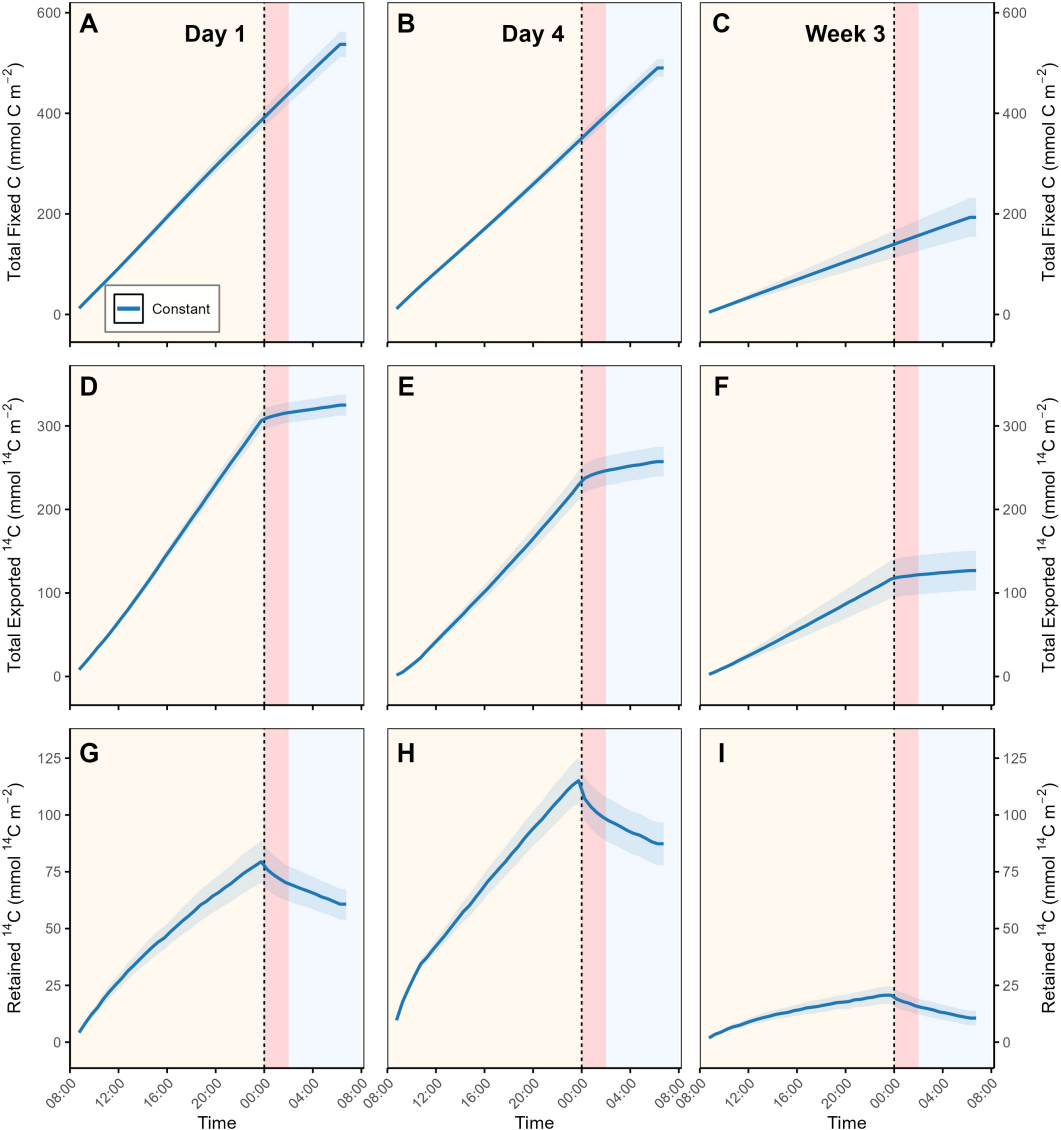
Total fixed, exported, and retained ¹^4^C for the Constant treatment on Day 1 **(A, D, G)**, Day 4 **(B, E, H)**, and Week 3 **(C, F, I)**. Total Fixed C **(A–C)** (mmol C m^-^²), Total Export C **(D–F)** (mmol C m^-^²), and Retained C in the source leaf **(G–I)** (mmol C m^-^²) are shown over the same 24-h period for the Constant lighting regime. The *x*-axis is expressed as clock time from 08:00 to 08:00 the following day (0–24 h). Columns correspond to Day 1 (left), Day 4 (middle), and Week 3 (right). Total quantities were obtained by numerically integrating the instantaneous NCER and export rates and converting to mmol C m^-^². Lines are hourly means (*n* = 4) ± SE. Background shading indicates the 16-h ^14^CO_2_ feeding period (light yellow, 0–16 h after lights-on), the initial 2 h nighttime chase window (red, 16–18 h), and the remaining 6-h chase period (light blue, 18–24 h). The vertical dashed line marks the end of the ^14^CO_2_ feed (16 h).

To test whether continuous light induces a carbon surplus that could contribute to feedback regulation, we analyzed the Constant treatment across acclimation using steady-state ¹^4^CO_2_ labeling during the daytime (Day 1, Day 4, and Week 3; **Figures 2**, **3**). Day 1 represents the initial condition prior to meaningful photoperiod-extension exposure, Day 4 represents a pre-injury stage (no visible chlorosis under our conditions), and Week 3 represents long-term exposure with severe photoperiodic injury.

Across the isotopic-equilibrium window (1.5–16 h), Constant Day 4 showed only a modest reduction in NCER relative to Day 1 (6.17 ± 0.06 vs. 6.91 ± 0.08 µmol CO_2_ m^-^² s^-^¹), whereas concurrent export was reduced more strongly (4.22 ± 0.08 vs. 5.52 ± 0.06 µmol CO_2_ m^-^² s^-^¹). This disproportionate reduction lowered relative export (68.25% ± 0.89% vs. 79.98% ± 0.55%, Day 4 vs. Day 1) and yielded a pattern in which total exported carbon declined substantially (~25%) while total fixed carbon declined more modestly (~10%), resulting in a markedly larger retained pool at the end of the feed on Day 4 (+~45%) despite lower NCER and lower cumulative assimilation (**Figures 2**, **3**). Conceptually, this response matches steady-state ¹^4^CO_2_ observations in other contexts where assimilation and export become partially uncoupled, such that leaves retain a larger fraction of newly fixed carbon when export does not scale proportionally with photosynthesis (Grodzinski et al., 1998; Leonardos and Grodzinski, 2000).

Partitioning of retained ¹^4^C after 24 h into soluble (sucrose-dominant) and insoluble (starch-dominant) fractions indicated broadly similar excess contributions under continuous light (**Supplementary Figure 1**), consistent with an overall shift toward retention rather than rerouting into a single dominant storage pool. However, this protocol does not localize the causal step (e.g., altered phloem loading capacity, increased transport resistance, or reduced sink demand), which would require denser destructive time series and transport-focused measurements (Ainsworth and Bush, 2011; Paul and Foyer, 2001).

### 3.2 Continuous light alters diel phase relationships and decouples carbon assimilation from water loss

**Figure 4:**
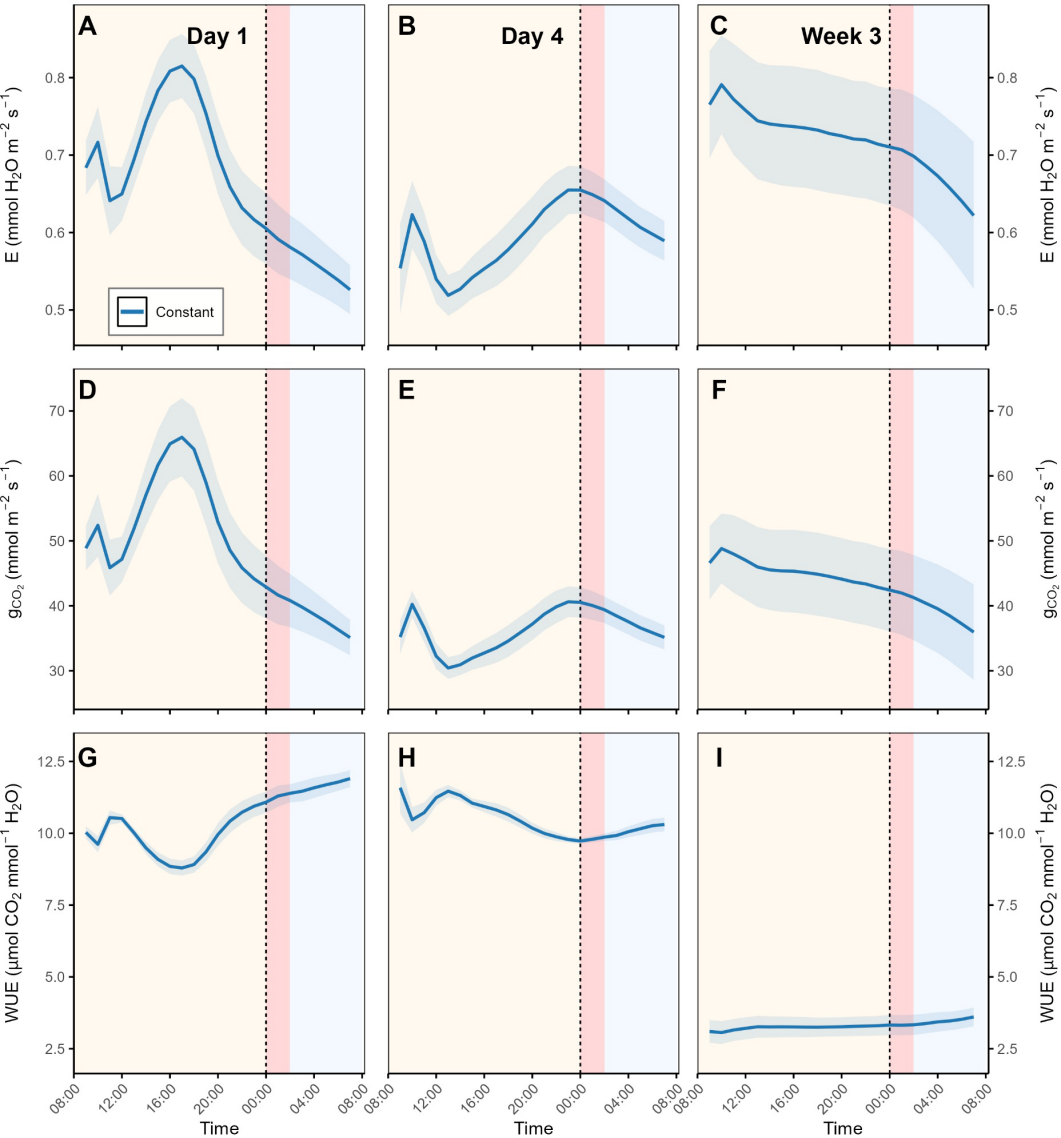
Instantaneous transpiration (E), stomatal conductance to CO_2_ (gCO_2_), and water-use efficiency (WUE) for Constant-light plants during the daytime ¹^4^CO_2_ labeling cycle on Day 1, Day 4, and Week 3. Panels **(A–C)** show E, panels **(D–F)** show gCO_2_, and panels G–I show WUE, with columns corresponding to Day 1, Day 4, and Week 3, respectively. The *x*-axis is expressed as clock time from 08:00 to 08:00 the following day (0–24 h). Lines are half-hourly means (*n* = 4) ± SE. Background shading indicates the 16-h ¹^4^CO_2_ feeding period (light yellow, 0–16 h after lights-on), the initial 2 h nighttime chase window (red, 16–18 h), and the remaining 6-h chase period (light blue, 18–24 h). The vertical dashed line marks the end of the ¹^4^CO_2_ feed (16 h).

Because we observed shifts in diel timing between Day 1 and Day 4 under continuous light, we quantified phase shifts (acrophase) for carbon and water-flux variables (**Figures 2**, **4**; **Supplementary Table 1**). On Day 4, peak NCER was delayed by +9.503 ± 0.847 h relative to Day 1 (*p* < 0.01), while peak export was delayed by +5.927 ± 0.580 h (*p* < 0.01), indicating that export phase-shifted less than photosynthetic carbon uptake. Transpiration (E) also shifted markedly (+8.600 ± 0.632 h; *p* < 0.001) and decreased in the peak window (−0.154 ± 0.039; *p* < 0.05), with stomatal indicators shifting in parallel (gCO_2_: +8.915 ± 0.731 h, *p* < 0.01; C_i_: +8.565 ± 0.790 h, *p* < 0.01). Collectively, these results indicate that early continuous-light exposure manifests primarily as loss of normal diel coordination, with non-uniform phase shifts among NCER, export, and transpiration.

By Week 3, when injury was severe, carbon fluxes under Constant were strongly suppressed relative to Day 1: NCER declined by 64.4% ± 1.8% and concurrent export declined by 61.7% ± 2.0%, producing markedly smaller daytime labeled pools at the end of the 16-h feed. Total fixed C was 64.4% ± 7.1% lower (385.69 ± 17.98 vs. 137.44 ± 26.70 mmol CO_2_ m^-^²), total exported C was 61.9% ± 7.6% lower (306.24 ± 12.34 vs. 116.77 ± 22.79 mmol CO_2_ m^-^²), and total retained C was 74.0% ± 5.9% lower (79.45 ± 9.28 vs. 20.67 ± 4.04 mmol CO_2_ m^-^²). Despite depressed absolute pools, relative export was higher at Week 3 (80.0% ± 0.55% vs. 85.83% ± 0.63%), which should be interpreted cautiously given the collapse in absolute carbon assimilation. In contrast to carbon assimilation, transpiration was not similarly reduced and was 4.24% ± 3.13% higher (0.708 ± 0.013 vs. 0.738 ± 0.017 mmol H_2_O m^-^² s^-^¹, Day 1 vs. Week 3), yielding a pronounced −67.17% ± 0.91% decline in WUE (9.89 ± 0.12 vs. 3.25 ± 0.08 mmol CO_2_ mol^-^¹ H_2_O).

### 3.3 Dynamic 24-h LED recipes sustained long-term carbon balance and water-use efficiency

**Figure 5:**
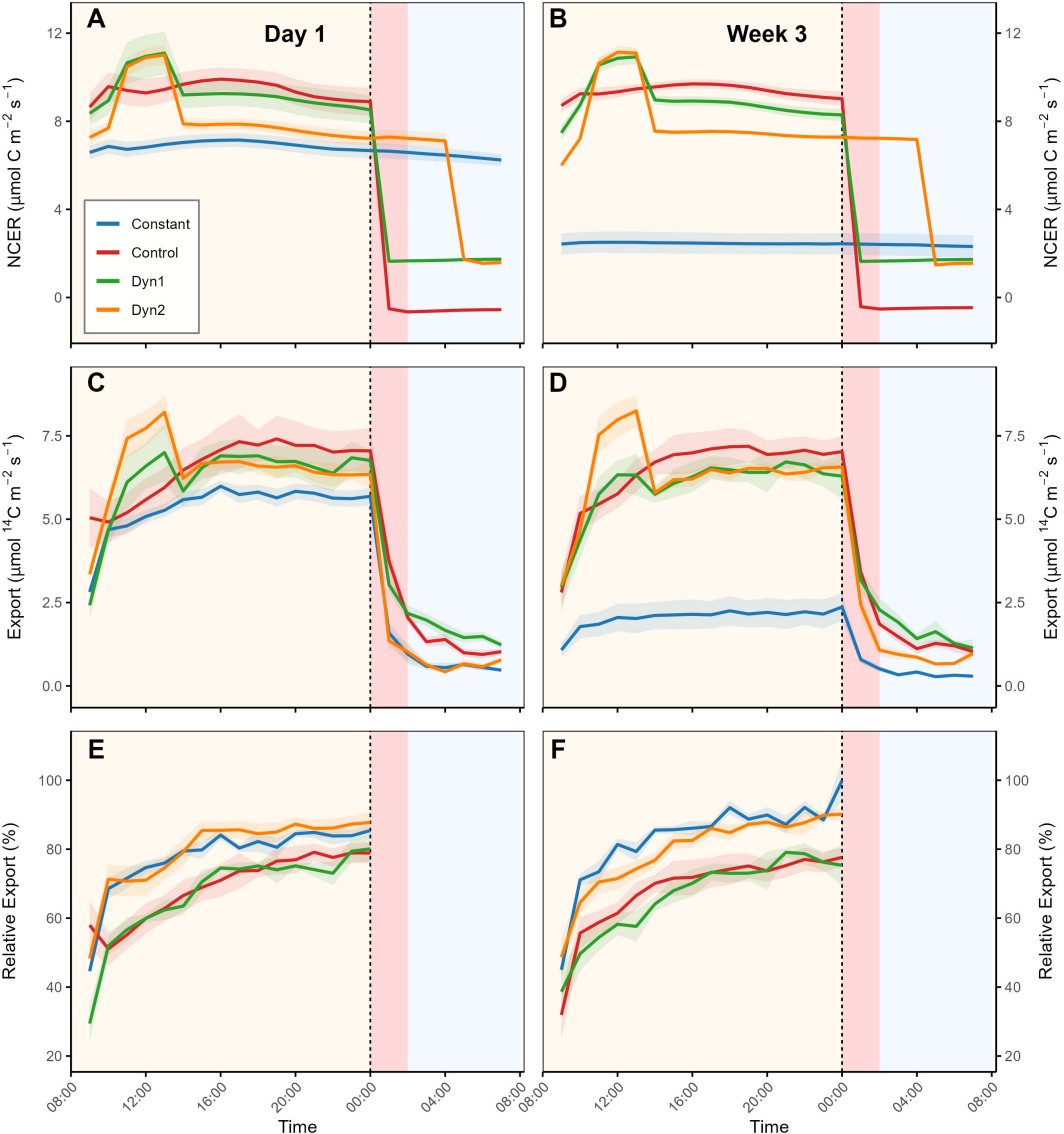
Diel carbon fluxes across light regimes on Day 1 vs. Week 3. Panels are arranged by day; Day 1 **(A, C, E)**, Week 3 (B, D, F) and variable; NCER **(A, B)** (µmol C m^-^² s^-^¹), ¹^4^C export rate **(C, D)** (µmol C m^-^² s^-^¹), and relative export **(E, F)** (%, plotted only when label was present) for Control (red), Constant (blue), Dynamic 1 (green), and Dynamic 2 (orange). The *x*-axis is expressed as clock time from 08:00 to 08:00 the following day (0–24 h). Labeling was daytime labeled (¹^4^CO_2_ present for the first 16 h, then unlabeled chase for the remaining 8 h), indicated by a background shading of yellow. Note that, during chase, the calculation of export no longer depends on NCER as there is no concurrent export; instead, it is calculated as the loss of retained labeled C over time. The lack of labeling during the chase results in a drastic drop in export. Lines are hourly means (*n* = 4) ± SE. Background shading indicates the 16-h ^14^CO_2_ feeding period (light yellow, 0–16 h after lights-on), the initial 2 h nighttime chase window (red, 16–18 h), and the remaining 6-h chase period (light blue, 18–24 h). The vertical dashed line marks the end of the ^14^CO_2_ feed (16 h). Also, note that relative exportduring the nighttime chase period is not shown as it uses a different formula that normalizes to each plant’s retained pool at 16 h and is termed remobilizationefficiency (**Table 5**; **Supplementary Table 3**).

**Figure 6:**
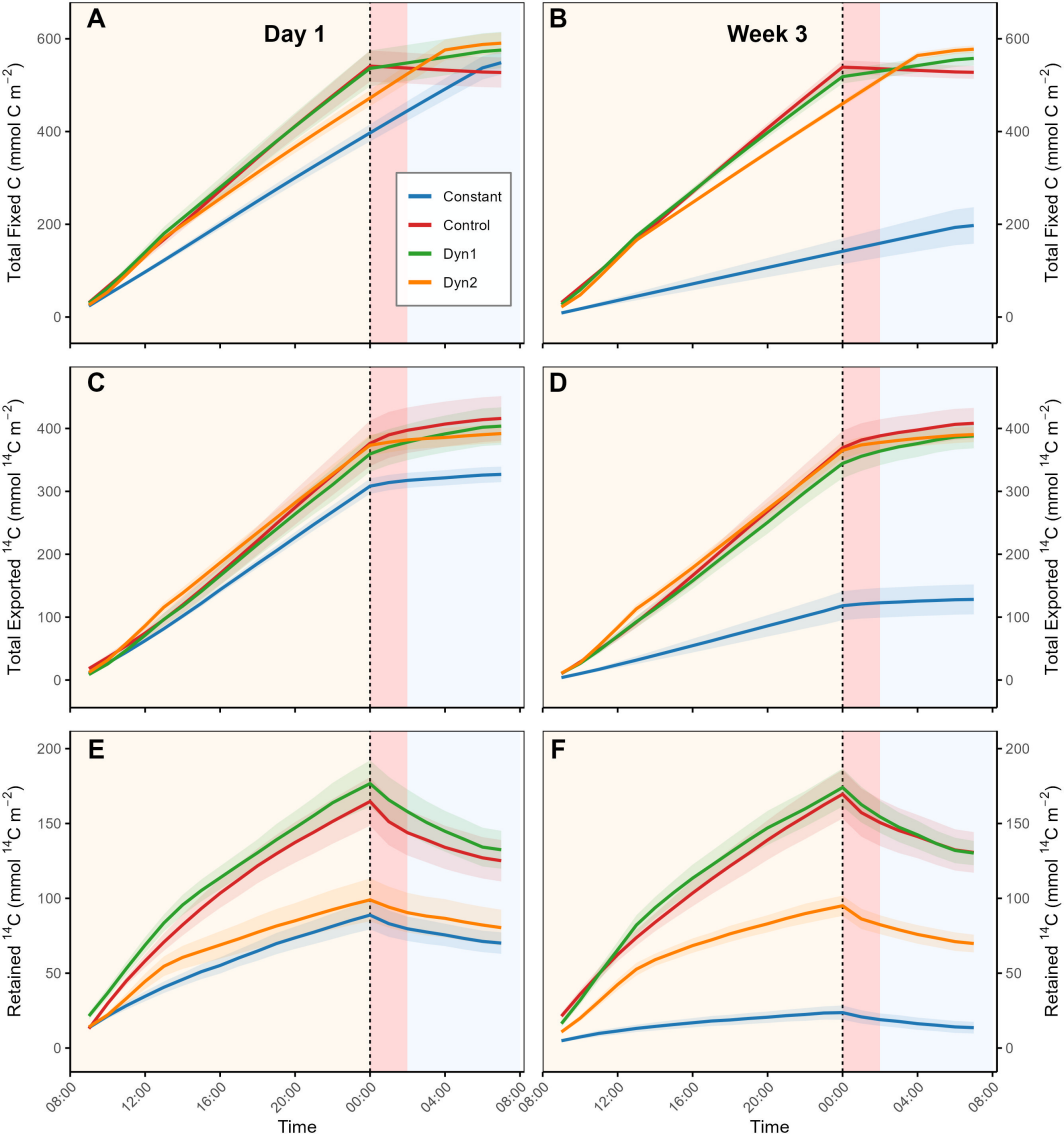
Total carbon budgets across light regimes on Day 1 vs. Week 3. Panels show Total Fixed C **(A, B)** (mmol C m^-^²), Total Export C **(C, D)** (mmol C m^-^²), and Retained C in the source leaf **(E, F)** (mmol C m^-^²) for Control (red), Constant (blue), Dynamic 1 (green), and Dynamic 2 (orange). The *x*-axis is expressed as clock time from 08:00 to 08:00 the following day (0–24 h). Lines are hourly means (*n* = 4) ± SE. Background shading indicates the 16-h ^14^CO_2_ feeding period (light yellow, 0–16 h after lights-on), the initial 2 h nighttime chase window (red, 16–18 h), and the remaining 6-h chase period (light blue, 18–24 h). The vertical dashed line marks the end of the ^14^CO_2_ feed (16 h).

After 3 weeks, neither Dynamic recipe displayed visible photoperiodic injury. To test whether subtler dysfunction occurred, we compared steady-state ¹^4^CO_2_-derived variables (export, relative export, total exported C, and retained C) between Day 1 and Week 3 across treatments (Control, Constant, Dynamic 1, and Dynamic 2) over the labeled photoperiod window (1.5–16 h; **Figures 5**, **6**; **Supplementary Figure 2**; **Supplementary Table 2**), using the same steady-state labeling framework used to quantify immediate export under different environments (Grodzinski et al., 1998; Lanoue et al., 2018).

As a negative control, Constant total exported C declined sharply from Day 1 (289 ± 12 mmol C m^-^²) to Week 3 (109 ± 22 mmol C m^-^²; *p* < 0.01), consistent with severe impairment under injury. In contrast, no significant acclimation-related change in total exported C was detected in Control (344 ± 32 vs. 347 ± 26 mmol C m^-^²), Dynamic 1 (330 ± 27 vs. 321 ± 22 mmol C m^-^²), or Dynamic 2 (349 ± 16 vs. 343 ± 12 mmol C m^-^²) (all *p* > 0.5; **Supplementary Table 2**). No significant acclimation-related change was detected in other ¹^4^C-derived variables for Control or either Dynamic treatment.

**Figure 7:**
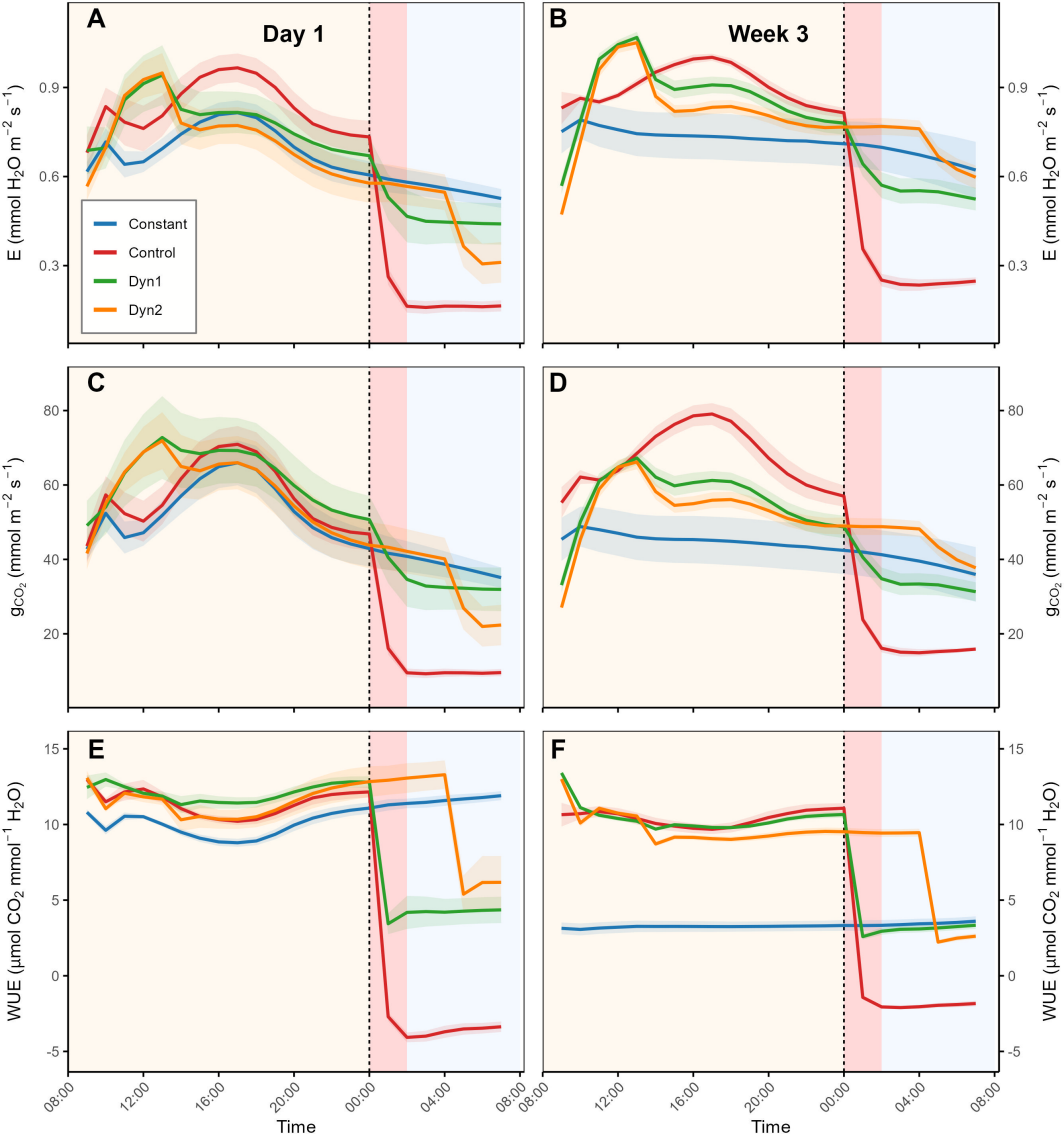
Diel water-use variables across light regimes on Day 1 vs. Week 3. Panels show transpiration, E **(A, B)** (mmol H_2_O m^-^² s^-^¹), stomatal conductance (g_CO_2__) **(C, D)** (mmol m^-^² s^-^¹), and water-use efficiency (WUE) [**(E, F)**; µmol CO_2_ mmol^-^¹ H_2_O] for Control (red), Constant (blue), Dynamic 1 (green), and Dynamic 2 (orange). The *x*-axis is expressed as clock time from 08:00 to 08:00 the following day (0–24 h). Labeling was daytime labeled (¹^4^CO_2_ present for the first 16 h, then unlabeled chase for the remaining 8 h), indicated by a background shading of yellow. The 16-h photoperiod is marked with a vertical dotted line for clarity. Nighttime chase is divided into two windows, 16–18 h (red) and 18–24 h (blue). Lines are means ± SE (*n* = 4).

To evaluate integrated carbon–water performance, we analyzed Week 3 whole-day (0–24 h) integrated WUE (**Figure 7**; **Table 2**). Including all treatments, WUE differed strongly among lighting regimes (one-way ANOVA, *p* ≈ 2.7×10^-9^), driven by Constant (3.29 ± 0.35) being markedly lower than Control and both Dynamic schedules (Control: 9.04 ± 0.28; Dynamic 1: 8.74 ± 0.14; Dynamic 2: 8.95 ± 0.18; Tukey HSD: Constant< Control = Dynamic 1 = Dynamic 2). Excluding Constant, WUE_{int} did not differ among Control, Dynamic 1, and Dynamic 2 at Week 3 (*p* = 0.327), indicating that time-partitioned spectra did not impose a detectable penalty on integrated WUE under DLI-matched conditions (**Table 2**).

**Table 2:** Whole-day water-use efficiency after 3 weeks of acclimation.

Treatment	Total fixed C (mmol m^-^² day^-^¹)	Total transpiration (mmol m^-^² day^-^¹)	Whole-day WUE (mmol mol^-^¹)
Control	519.7 ± 13.6	57.51 ± 0.70	9.04 ± 0.28a
Dynamic 1	553.1 ± 15.2	63.37 ± 2.62	8.74 ± 0.14a
Dynamic 2	574.1 ± 8.0	64.22 ± 1.87	8.95 ± 0.18a
Constant	197.3 ± 39.3	58.18 ± 6.25	3.29 ± 0.35b

Including Constant: WUE_int_ [F(3,12) = 31.7, p = 2.67×10^-9^]. Excluding Constant: WUE_int_ [F(2,9) = 1.19, p = 0.327].

Whole-day integrated water-use efficiency (0–24 h) was calculated from gas-exchange time series as ${\text{WUE}}_{\mathrm{int}}=\frac{{\text{NCER}}_{0–24}}{{\text{E}}_{0–24}}$ (mean ± SE; n = 4 biological replicates per treatment). A one-way ANOVA across all Week 3 treatments (Control, Constant, Dynamic 1, and Dynamic 2) detected a strong treatment effect on whole-day WUE, and Tukey HSD indicated Constant< Control = Dynamic 1 = Dynamic 2 (denoted by letters, α = 0.05)^1^. Since Constant represents an injury-associated continuous-light condition, the analysis was repeated, excluding Constant to test the hypothesis among non-injured treatments (Control, Dynamic 1, and Dynamic 2), which also showed no treatment effect^2^.

### 3.4 A transient blue-enriched peak did not reprogram daytime relative export

To isolate the effect of a transient 3-h blue-enriched peak (to 390 µmol m^-^² s^-^¹, total PPFD) on instantaneous carbon balance, we analyzed Day 1 responses in three time windows: baseline (1.5–2.0 h), peak (3.0–5.0 h), and post (6.0–8.0 h). A linear mixed event study of relative export detected strong time-window structure and an overall treatment effect, but no Treat × Window interaction, indicating broadly parallel temporal patterns among treatments (**Table 3**). Baseline relative export differed by baseline light level: Constant and Dynamic 2 (baseline 200 µmol m^-^² s^-^¹) were ~70% export, whereas Control (300) and Dynamic 1 (250) were ~55% export. A planned baseline contrast confirmed this separation: (Control + Dynamic 1)/2 − (Constant + Dynamic 2)/2 = −14.75 ± 3.68%, *p* = 0.00044. Planned difference-in-differences contrasts indicated that the blue-enriched peak did not measurably alter relative export relative to the static references (**Table 4**). Baseline relative export (“morning setpoint”) strongly predicted later relative export across the remaining photoperiod (Pearson *r* = 0.905, *p* = 1.47×10^-6^), consistent with the visual grouping of Control with Dynamic 1 and Constant with Dynamic 2 (**Figure 5E**).

**Table 3:** Event-study mixed model estimated relative export by treatment and time-windows before, during, and after a 3-h blue-enriched peak on Day 1.

Treatment	Baseline relative export (1.5–2.0 h; 09:30–10:00)	Peak relative export (3.0–5.0 h; 11:00–13:00)	Post relative export (6.0–8.0 h; 14:00–16:00)
Constant	69.77 ± 3.68	75.32 ± 3.14	81.94 ± 3.14
Control	55.79 ± 3.68	61.36 ± 3.14	69.95 ± 3.14
Dynamic 1	55.48 ± 3.68	61.15 ± 3.14	72.54 ± 3.14
Dynamic 2	71.00 ± 3.68	72.78 ± 3.14	85.44 ± 3.14

Treatment F-statistic (3, 12.31) = 7.08, p = 0.00512; Time-window F-statistic (4, 176) = 78.12, p = 5.57×10^-^³^8^; Treat × Time F-statistic (12, 176) = 1.31, p = 0.216.

Reported in estimated marginal means and standard error (n = 4), alpha = 0.05.

**Table 4:** Planned contrasts (estimate ± SE) testing a 3-h blue-enriched peak perturbation and baseline separation in relative export.

Difference-in-differences contrasts	Comparison	Estimate ± SE (%)	Df	t	p
Δ (peak − baseline)	Dynamic 1 − Control	0.09 ± 3.51	176.00	0.03	0.9787
Δ (peak − baseline)	Dynamic 2 − Constant	−3.77 ± 3.51	176.00	−1.07	0.2842
Δ (post − baseline)	Dynamic 1 − Control	2.89 ± 3.51	176.00	0.82	0.4106
Δ (post − baseline)	Dynamic 2 − Constant	2.26 ± 3.51	176.00	0.65	0.5196

### 3.5 Subjective night ¹^4^C remobilization was conserved under both static and dim-night photoperiod extensions

To quantify mobilization and translocation of assimilates accumulated during the preceding labeled photoperiod (1.5–16 h), which is conceptually distinct from concurrent export during the feed, we measured whole-night chase export (16–24 h) and calculated whole-night “remobilization efficiency” as: $\text{Remobilization efficiency}=\frac{{\text{Exported C}}_{16-24}}{{\text{Retained C}}_{1.5-16}}\times 100\%$ (**Figures 5**, **6**; **Table 5**). We also subdivided the night into 16–18 h and 18–24 h to capture distinct remobilization kinetics (**Supplementary Table 3**). Across all replicates, ~41% of whole-night remobilization occurred in 16–18 h and ~59% occurred in 18–24 h, consistent with classic radiotracer evidence for biphasic export in which early night export draws strongly on soluble pools while later export increasingly depends on starch mobilization (Fondy and Geiger, 1982; Stitt and Zeeman, 2012).

**Table 5:** Whole-night (16–24 h) chase carbon remobilization at Day 1 and Week 3 calculated as Whole-night remobilization = $\frac{{\text{Exported C}}_{16–24}}{{\text{Retained C}}_{1.5–16}}$ × 100%.

Treatment	Total exported C (mmol C m^-^²) (16–24 h)	Retained C (mmol C m^-^²) (1.5–16 h)	Remobilization efficiency (%)
Day 1			
Control	36.52 ± 3.02	137.00 ± 15.16	27.12 ± 1.82
Dynamic 1	38.89 ± 3.24	142.36 ± 12.10	27.30 ± 1.66
Dynamic 2	21.80 ± 3.46	78.02 ± 12.70	20.93 ± 1.64
Constant	17.24 ± 2.97	68.41 ± 7.84	24.87 ± 2.70
Week 3			
Control	36.38 ± 2.92	135.64 ± 13.64	27.04 ± 1.24
Dynamic 1	42.30 ± 4.82	143.53 ± 10.37	29.20 ± 1.25
Dynamic 2	24.23 ± 2.36	76.80 ± 5.38	31.68 ± 2.87
Constant	9.44 ± 0.74	16.73 ± 3.17	60.45 ± 7.43

^1^ Acclimation F(df = 1,6): Control F(1,6) = 0.00, p = 0.972; Dynamic 1 F(1,6) = 0.13, p = 0.729; Dynamic 2 F(1,6) = 5.33, p = 0.060; Constant F(1,6) = 20.25, p = 0.0041.

^2^ Day 1 (Control, Constant, Dynamic 1, Dynamic 2) F(3,12) = 1.86, p = 0.190.

^3^ Week 3 including Constant F(3,12) = 14.79, p = 2.47×10^-4^; Week 3 excluding Constant (Control, Dynamic 1, Dynamic 2 only) F(2,9) = 1.42, p = 0.290.

Values are mean ± SE (n = 4 plants per treatment × stage). A one-way ANOVA was performed to compare acclimation (Day 1 vs. Week 3) within each treatment for remobilization efficiency^1^. One-way ANOVAs were also performed for within-day treatment comparisons on Day 1^2^ and Week 3^3^.

Across treatments, whole-night remobilization efficiency was ~30% and was broadly conserved under Dynamic treatments relative to the dark-night Control on both Day 1 and Week 3 (**Table 5**). Treatment effects were not detected in most chase windows. A transient difference occurred on Day 1 in the late-night window (18–24 h), where remobilization efficiency was higher in Dynamic 1 than in Dynamic 2, while Control and Constant were intermediate and not distinguishable from either Dynamic treatment. By Week 3 (excluding Constant), remobilization efficiency did not differ among Control, Dynamic 1, and Dynamic 2 in any chase window (all *p* ≥ 0.286). Overall, whole-night remobilization scaled positively with the retained pool size across treatments and acclimation stages (except Constant Week 3) (**Supplementary Figure 3**).

Upon seeing this result, we wanted to see if the higher retained pool under Constant Day 4 compared to Constant Day 1 affected remobilization efficiency or if it would instead follow the general positive scaling pattern observed in the other treatments. In Constant, remobilization efficiency did not differ between Day 1 (24.87% ± 2.70%) and Day 4 (26.75% ± 2.85%), indicating that proportional remobilization of previously fixed carbon remained coupled to retained pool size over the first 4 days of continuous light exposure.

### 3.6 Estimating remobilization contributions in addition to concurrent export using nighttime steady-state ¹^4^CO_2_ labeling

To evaluate export behavior on the first day as plants crossed the entrained 16-h photoperiod boundary while label was still being assimilated, we supplied ¹^4^CO_2_ continuously throughout the final 16 h of the 24-h cycle and termed the protocol “nighttime labeling” with no chase (**Figure 7**; **Supplementary Figure 4**). Under Constant, relative export remained stable across the night at ~80% in all time-matched windows, consistent with sustained coupling between assimilation and export under steady irradiance (Geiger and Fondy, 1979). Under Dynamic 1 and Dynamic 2, relative export increased above 100% only after each treatment recipe entered the dim blue plus far-red phase, indicating that export exceeded assimilation and therefore acquired a contribution from previously fixed carbon pools during those dim windows (**Figure 7**).

To conservatively estimate the remobilized component after each transition, we summed net export only when it exceeded assimilation during each Dynamic treatment’s respective transition to dim light (16 h or 20 h) to the end of the run (24 h): Estimated remobilization_transition–end_ = (Exp − NCER)_transition–end_ (when Exp > NCER). Across replicates pooled across Dynamic 1 and Dynamic 2 (*n* = 8), the pre-transition retained labeled pool strongly predicted estimated remobilization of labeled C after transition (slope = 0.1044; *R*² = 0.946; *p* = 5.0×10^-5^) (**Supplementary Figure 5**). Note that the labeled fraction of C being remobilized is an underestimate because we did not label during the first half of the photoperiod (labeled C is diluted with unlabeled C); therefore, it is only used as semi-quantitative evidence wherein remobilization occurred in addition to concurrent export.

## 4 Discussion

### 4.1 Early continuous light induces a source-leaf “retention phenotype” consistent with rapid uncoupling of assimilation and concurrent export

By the fourth day of continuous light exposure, prior to visible injury, leaves retained substantially more newly fixed carbon because concurrent export declined more strongly than NCER, lowering relative export and enlarging the retained pool (**Figures 2**, **3**). This “retention phenotype” aligns with the broader concept that carbon assimilation and export are separable, regulated processes, and can become partially uncoupled even when steady-state photosynthesis is only modestly affected (Ainsworth and Bush, 2011; Paul and Foyer, 2001). Consistent with this view, steady-state ¹^4^CO_2_ studies in multiple contexts have shown that leaves can retain proportionately more recently fixed carbon when concurrent export does not scale proportionally with assimilation, including under short-term CO_2_ enrichment and after acclimation to low temperature (Grodzinski et al., 1998; Leonardos and Grodzinski, 2000; Leonardos et al., 2003).

In the continuous-light tomato literature, injury is often accompanied by carbohydrate accumulation and suppressed photosynthetic performance (Demers et al., 1998; Haque et al., 2015; Sysoeva et al., 2010), with evidence that sucrose/starch levels can correlate negatively with PSII maximum quantum efficiency under abnormal light/dark cycles and continuous light (Velez-Ramirez et al., 2017a). The present short-term continuous light exposure results refine this narrative by showing that concurrent export (and thus relative export) was more sensitive than NCER, producing elevated carbon retention before the later collapse in carbon assimilation observed after long-term exposure. This supports the conservative interpretation that increased retention is an early consequence of continuous light exposure and is consistent with an emerging assimilation–concurrent export decoupling under extended photoperiods, without implying that carbohydrate accumulation is the primary cause of chlorosis (Sysoeva et al., 2010; Velez-Ramirez et al., 2011).

A central mechanistic question is whether this early retention phenotype reflects (i) a generalized downstream limitation in transport/loading and sink utilization, or (ii) a shift in source-side end-product commitment/partitioning during assimilation. A useful diagnostic prediction is that a shared downstream limitation in phloem loading/transport and/or sink utilization should constrain export regardless of carbon origin and thus would be expected to depress both concurrent export of newly assimilated carbon during the ¹^4^CO_2_ feed and export supported by previously assimilated (and stored) pools during the post-label chase. In contrast, a source-leaf limitation expressed during active assimilation (e.g., altered end-product commitment and Pi-linked regulation) could selectively reduce concurrent export coupling while leaving chase-derived export largely proportional to retained labeled carbon pool size. Earlier hypotheses in tomato extended-photoperiod work proposed that reduced sucrose commitment capacity, often discussed in relation to SPS regulation, can promote a Pi-limited state, leading to accumulation of carbohydrates and phosphorylated intermediates and biasing carbon toward starch (Ayari et al., 2000; Dorais et al., 1996; Huber and Huber, 1992, 1996). This aligns with the broader triose-phosphate utilization (TPU)/Pi feedback framework, in which net assimilation and partitioning are constrained by end-product synthesis and Pi recycling capacity rather than by light harvesting *per se* (McClain and Sharkey, 2019; Yang et al., 2016). In that framework, a key limitation may be found at the chloroplast–cytosol exchange, where reduced cytosolic Pi availability can constrain Pi-coupled triose-phosphate export from the chloroplast, increasing reliance on starch buffering as an alternative sink (McClain and Sharkey, 2019; Walters et al., 2004). Starch synthesis is itself sensitive to the 3-phosphoglycerate:Pi balance via allosteric regulation of ADP-glucose pyrophosphorylase, providing a biochemical route by which altered Pi economy can bias retention toward starch without requiring an initial defect in light capture (Kleczkowski, 1999; Walters et al., 2004). Thus, reduced relative export under short-term continuous light can be interpreted as a regulated shift in end-product commitment and partitioning (a source-side constraint) without requiring early failure of long-distance transport (Ayari et al., 2000; McClain and Sharkey, 2019).

Importantly, our post-label chase analysis (Section 5.5) provides an internal constraint on early shared sink/transport bottleneck interpretations: export supported by previously assimilated/labeled pools during the post-label ^14^C chase interval remained proportional to their respective retained pool sizes (including in Constant Day 4) (expressed as remobilization efficiency in **Table 5**; **Supplementary Figure 3**), indicating that chase-derived export was not measurably suppressed at this stage. This pattern is more consistent with a selective disruption of assimilation–concurrent export coupling during the feed than with a generalized inability to export carbon out of the leaf.

### 4.2 Phase disruption and carbon–water decoupling support circadian asynchrony as an early signature of continuous-light stress

An additional phenotype was observed after 4 days of continuous light, and leaves displayed large phase delays across NCER, export, and transpiration, with non-uniform shifts among these processes (**Figure 4**; **Supplementary Table 1**). This pattern is consistent with historical and contemporary evidence that tomato is sensitive to abnormal photoperiod timing and that photoperiodic injury is linked to disrupted circadian coordination rather than continuous light *per se* (Highkin and Hanson, 1954; Hillman, 1956; Velez-Ramirez et al., 2017a). A mechanistic precedent for such uncoupling comes from circadian studies in *Arabidopsis*: the ZEITLUPE photoreceptor mutant showed partially independent circadian regulation of CO_2_ assimilation and stomatal conductance, indicating that guard cell and mesophyll rhythms can be differentially regulated and that coordination among physiological oscillators is not guaranteed even within a single leaf (Dodd et al., 2004). More broadly, correct matching of internal circadian period to the external cycle increases carbon assimilation and growth, underscoring the productivity cost of a temporal mismatch (Dodd et al., 2005). At higher scales of complexity, under continuous light, coupling between organs can weaken (e.g., root–shoot desynchrony), and sugar/photosynthesis-associated signals can modulate oscillator behavior across tissues, providing plausible routes by which carbon acquisition, allocation, and water relations can become misaligned across the whole plant (Dodd et al., 2015; Haydon et al., 2013).

In tomato, evidence that tolerance and sensitivity to continuous light can be influenced by grafting and rootstock–scion interactions is consistent with whole-plant signaling contributing to injury expression, supporting the plausibility that organ-level desynchrony and altered systemic signaling influence how continuous light is integrated across source and sink tissues (Lanoue et al., 2021; Velez-Ramirez et al., 2015). In this context, the Day 4 phase divergence between NCER and export is notable because it suggests that source-leaf carbon uptake and the processes that determine carbon delivery away from the leaf (export and associated sink demand) can become temporally misaligned before visible chlorosis develops. Such timing disruption provides a mechanistic bridge between continuous-light circadian entrainment hypotheses (Marie et al., 2022) and the early retention phenotype described in Section 5.1: if periods of high assimilation become out of phase with periods of high sucrose-commitment/export capacity (and associated sink utilization), then relative export can decline even without an immediate collapse in photosynthetic capacity (Dodd et al., 2005; Haydon et al., 2013; Velez-Ramirez et al., 2017a). Together, these frameworks support interpreting the Day 4 timing shifts as an early transition toward circadian asynchrony that precedes, and may mechanistically enable, the stronger carbon–water decoupling observed under long-term injury.

### 4.3 Dynamic LED recipes likely mitigate injury by stabilizing integrated carbon balance and preserving diel coordination of export

**Figure 8:**
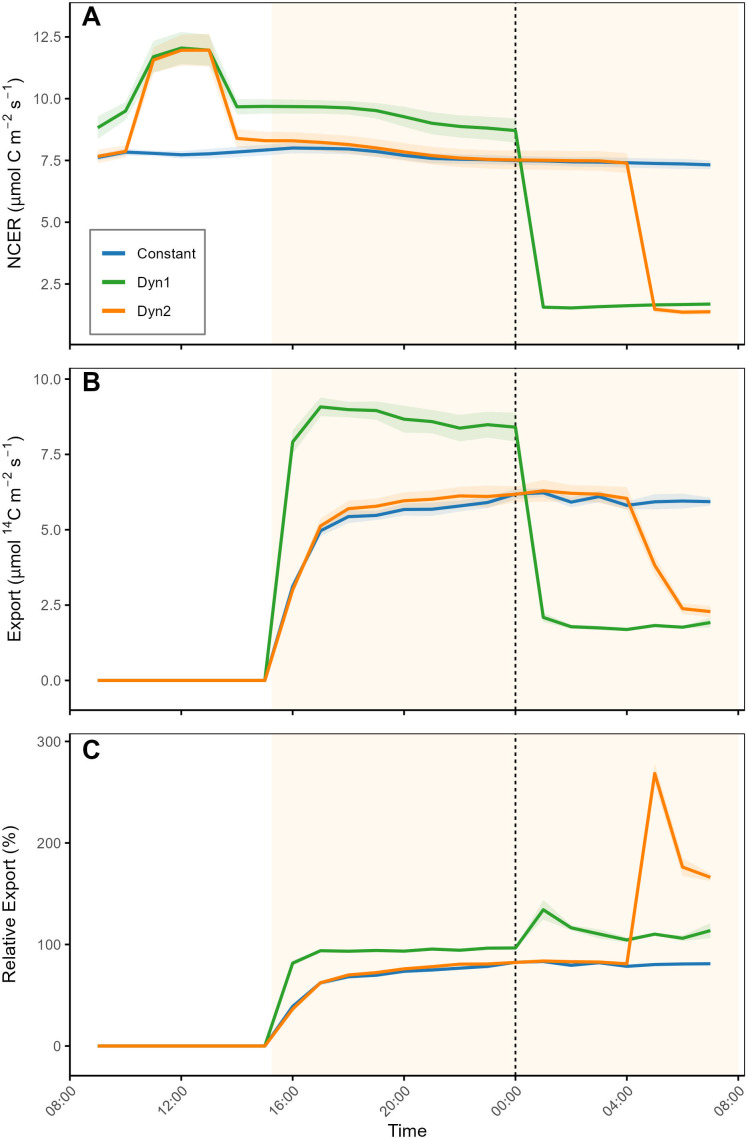
Nighttime labeled ^14^C feed showing the net carbon exchange rate (NCER) **(A)** µmol C m^-^² s^-^¹), ¹^4^C export rate **(B)** µmol C m^-^² s^-^¹), and relative export **(C)** ((export/NCER) x 100%) during the first day of treatments for Constant (blue), Dynamic 1 (green), and Dynamic 2 (orange). The *x*-axis is expressed as clock time from 08:00 to 08:00 the following day (0–24 h). ^14^CO_2_ was fed for 16 h starting midway through the photoperiod (~15:30) and continued throughout subjective nighttime (yellow shaded area). Tomato plants were previously entrained to 16-h photoperiods (noted as a dashed vertical line at 00:00). There was no chase period in this experiment. Lines are means ± SE (*n* = 4).

After 3 weeks, both Dynamic recipes maintained stable daytime carbon assimilation and concurrent export without detectable negative effects to whole-day integrated WUE relative to Control, whereas Constant showed a pronounced collapse in carbon assimilation and export alongside a major decline in WUE (**Figures 5**-**8**; **Table 2**; **Supplementary Table 2**). This divergence aligns with the growing body of work showing that photoperiodic injury in tomato is not an unavoidable consequence of a 24-h photoperiod but depends strongly on whether the regime provides physiologically meaningful temporal cues via spectrum and/or irradiance structure (Hao et al., 2025; Lanoue et al., 2019, 2026; Marie et al., 2022, 2024). Dynamic regimes imposing strong day–night contrast permit injury-free tomato production under continuous lighting and can sustain yield and photochemical performance relative to control photoperiod (12 and 16 h) lighting at matched DLI (Lanoue et al., 2019, 2026). The present steady-state ¹^4^CO_2_ results add a carbon-allocation dimension to this framework by demonstrating that Dynamic regimes preserved long-term coupling between assimilation and export/retention behavior, whereas Constant developed severe impairment. Crucially, the Dynamic regimes were not merely “injury-free”, they also did not develop the early static continuous light Day 4 retention phenotype as they maintained stable daytime assimilation–concurrent export coupling over acclimation. They also maintained water relations in phase with carbon assimilation, a sign of circadian entrainment.

A key implication from the broader literature is that subjective-night spectral effects must be interpreted in the context of nighttime light intensity (“photon pressure”) and DLI, rather than as stand-alone determinants (Matsuda et al., 2016; Hao et al., 2025; Lanoue et al., 2019; 2026). These studies support a dose–context interpretation: subjective-night blue can be compatible with tolerance when delivered at sufficiently low PPFD within a strongly time-structured regime (such as when natural sunlight in greenhouses becomes a stronger driver in early spring), whereas higher-PPFD or blue-enriched subjective nights increase injury risk when temporal structure is weak (Hao et al., 2025; Lanoue et al., 2026; Matsuda et al., 2016). The simulated entrainment cues and 24-h differential in photon pressure we used for the Dynamic regimes (**Figure 1**; **Table 1**) proved to be an efficacious combination that satisfied both circadian rhythm and coordination of metabolism/photostasis.

Blue light is a plausible clock input because blue-light photoreceptors such as cryptochromes and the ZEITLUPE family participate in circadian entrainment and timing (Cashmore et al., 1999; Cashmore, 2003; Somers et al., 2000). At the mechanistic level, ZEITLUPE is a LOV-domain blue-light photoreceptor whose circadian function depends on its photochemistry and light-regulated control of clock protein stability; importantly, experimental and biochemical work emphasizes that ZEITLUPE can integrate blue-light fluence (time-integrated dose) and that its photocycle kinetics influence how the signal is “counted” over time (Pudasaini and Zoltowski, 2013; Pudasaini et al., 2017; Zoltowski and Imaizumi, 2014). This provides a plausible resolution for the apparent discrepancy between dim blue being helpful and higher blue being harmful (Lanoue et al., 2019; Matsuda et al., 2016). A long-duration dim blue phase can deliver substantial fluence and thus function as a timing cue with low photon pressure, whereas moderate-to-high nighttime blue simultaneously increases excitation pressure and strongly engages additional blue-light signaling pathways (notably cryptochrome 1), which have distinct fluence-rate sensitivities and phase-shifting effects on the circadian rhythm (Ponnu and Hoecker, 2022; Yu et al., 2010).

These photoreceptor-based entrainment ideas are consistent with the circadian-asynchrony trigger hypothesis for tomato injury (Velez-Ramirez et al., 2017a) and with the broader physiological interpretation that diel contrast can support photostasis under extended photoperiods (Marie et al., 2022, 2024). Proposed protective features of tolerant dynamic regimes include altered engagement of alternative electron sinks and redox-balancing pathways during the subjective night, including elevated photorespiration signatures and nighttime cyclic electron flow (Marie et al., 2024). These features are mechanistically compatible with the TPU/Pi feedback concepts discussed in Section 5.1 because sustained ATP turnover and energy balancing can reduce the likelihood of entering feedback states that decouple assimilation from end-product commitment and export (McClain and Sharkey, 2019; Yang et al., 2016). In parallel, spectral manipulations that alter phytochrome signaling, especially far-red enrichment and phytochrome A-mediated protection, reduce injury risk in tomato, supporting the plausibility that the far-red component of the dim night cue contributes via signaling and temporal organization rather than photon dose alone (Velez-Ramirez et al., 2019).

Photoreceptor signaling may also influence carbon allocation capacity more directly, offering a plausible link between recipe structure and sucrose-commitment control points without implying those mechanisms were measured here. Phytochrome-mediated regulation of SPS activity has been demonstrated in maize, indicating that SPS can, in principle, respond to red/far-red signaling inputs (Vassey, 1988). In tomato seedlings, intermittent blue supplementation under LED regimes increased SPS activity and SPS gene expression, supporting the plausibility that blue-enriched cues can modulate sucrose-commitment capacity (such as under the peak blue-enriched phase of our dynamic treatments) (Gao et al., 2025). Although such effects may depend strongly on developmental stage and background spectrum, these studies motivate the working hypothesis that dynamic 24-h schedules could mitigate injury partly by stabilizing diel control of sucrose commitment (e.g., SPS activation state) and chloroplast–cytosol exchange (Pi-coupled triose-phosphate export), in addition to their effects on the circadian rhythm.

### 4.4 A “morning setpoint” hypothesis: early-day conditions may constrain diel relative export trajectories more than transient midday perturbations

The Day 1 event study showed that a transient 3-h blue-enriched peak (390 µmol m^-^² s^-^¹), imposed ~2 h after photoperiod onset, did not measurably reprogram relative export compared to time-matched static references, whereas baseline relative export (the first 2 h of the photoperiod) strongly predicted each plant’s later-day relative export trajectory (**Figure 5E**; **Tables 3**, **4**). This finding builds on previous work in tomato that used similar steady-state ¹^4^CO_2_ labeling, where relative export was reported to be lower under treatments with higher assimilation (Lanoue et al., 2018). All treatments in Lanoue et al. (2018) followed a common increasing pattern across the photoperiod, which was also seen in our results, but we found that it was resilient to a strong light intensity/assimilation perturbation in the morning, which was not expected.

One interpretation is that early-day conditions prime a “setpoint” for the balance between sucrose commitment/export and temporary storage, and that a short-lived later perturbation may be insufficient to push the source leaf across the biochemical thresholds that trigger a different partitioning regime. This is consistent with the “overflow” hypothesis, in which starch accumulation is stimulated when photosynthetic carbon supply exceeds sucrose synthesis/export and/or sucrose storage capacity, including circumstances where Pi recycling and end-product synthesis become constraining (Huber and Huber, 1996; Nakai et al., 2023; Stitt, 1990). In strawberry, for example, starch production increased sharply when leaf sucrose exceeded an apparent storage-capacity threshold (~150 mmol C m^-^²), providing an explicit “capacity” point at which overflow to starch was recruited (Nakai et al., 2023). While the corresponding sucrose-capacity threshold is not yet defined for tomato source leaves in our system, the logic implies that a blue-rich PPFD pulse delivered early, before substantial sucrose accumulation and before any Pi/TPU-like feedback state develops, may increase instantaneous carbon input without forcing a proportional change in relative export if sucrose synthesis/export capacity is not yet saturated (Nakai et al., 2023; Stitt, 1990).

Mechanistically, this threshold framing is compatible with established control of sucrose synthesis and partitioning through Pi-linked regulation and sugar-status feedback. In the classic sucrose–starch control network, chloroplast triose-phosphate export is coupled to Pi counter-exchange, and sucrose synthesis must recycle Pi at a rate sufficient to support ongoing photosynthesis (Stitt, 1990). When sucrose accumulates, SPS can become downregulated and upstream hexose-phosphate pools increase; this is associated with increases in fructose-2,6-bisphosphate that inhibit cytosolic fructose-1,6-bisphosphatase, effectively shifting the “threshold” for triose-phosphate withdrawal and biasing partitioning toward starch (Huber and Huber, 1996; Stitt, 1990). Under this view, early-day differences in PPFD/NCER (and thus in the trajectory of sucrose build-up and Pi recycling demand) can establish different operating points that persist for hours, whereas a short pulse imposed later may not durably alter the regulatory state that determines the day’s relative export trajectory (Lanoue et al., 2018; Stitt, 1990). Importantly, the setpoint could be configured by sink demand/phloem loading capacity at the beginning of the day, in addition to source leaf capacity, which could make it very peculiar to the growing system/developmental stage used in a given study (Ainsworth and Bush, 2011; Paul and Foyer, 2001).

This synthesis yields testable predictions that refine the “morning setpoint” hypothesis. If setpoints reflect overflow/threshold dynamics, then (i) imposing the same high-PPFD/blue enrichment at dawn (before the setpoint is established) should shift the day-long relative export trajectory more strongly than imposing it mid-morning; (ii) imposing the perturbation late in the photoperiod, when sucrose pools and retained carbon are larger, should be more likely to recruit overflow-like retention (and potentially alter relative export) than the same perturbation early (Nakai et al., 2023; Stitt, 1990); and (iii) performing the same early/late timing of perturbations on homogeneous populations of plants with forced large and small sink demands (done with temperature of peripheral organs, girdling, single versus double leader, rest of canopy shaded versus lit, etc.). Because steady-state ¹^4^CO_2_ labeling quantifies concurrent assimilation and export continuously, it is well suited to map these hypothesized thresholds empirically in tomato by identifying when (during the day) changes in carbon input begin to elicit disproportionate changes in retention versus export, how large the retained pool must be to initiate overflow, and to discover any patterns in source/sink capacity/acclimation that may be used as a targetable genotypic trait or desirable phenotype in CEA. Should also alter retention.

### 4.5 Reserve-supported export is conserved across treatments, and dim night phases under dynamic LEDs add a concurrent export component

Across treatments, export supported by previously assimilated labeled carbon during an unlabeled chase (“remobilization efficiency”) remained comparable to the dark-night Control (**Table 5**). Notably, Constant also showed similar proportional remobilization of daytime labeled carbon on both Day 1 and Day 4 (**Supplementary Figure 3**). However, relative export was stable during the first night of static photoperiod extension, as monitored in the nighttime-labeled experiment (**Figure 7**), while it dropped after 4 days. Together, these observations infer that the pre-injury retention phenotype reflects selective impairment of assimilation–concurrent export coupling rather than sharing the limitation with remobilization that would occur at a downstream export pathway. Downstream loading/transport remains a plausible upper bound on export, conceptually including sucrose efflux to the apoplast via SUGARS WILL EVENTUALLY BE EXPORTED TRANSPORTERS followed by sucrose/H^+^ symport into the sieve element–companion cell complex via SUCROSE TRANSPORTER 1-type transporters (Chen et al., 2012; Hackel et al., 2006). Tomato-specific transporter localization control (e.g., SlSUT4 effects on SlSUT1 plasma membrane localization) remains a plausible regulatory node (Liang et al., 2023), but our evidence of remobilization scaling with the retained pool available (**Supplementary Figures 3**, **S5**) supports a pool-limited buffering mechanism.

It is interesting that export supported by previously assimilated carbon can remain coupled to retained pool size even without darkness. It means that there is continued access to rapidly mobilized soluble pools (including vacuolar sucrose buffering via tonoplast-localized SUCROSE TRANSPORTER 4-type sucrose/proton symporters) and a preserved late-window reserve contribution classically attributed in part to starch degradation (Fondy and Geiger, 1982; Schneider et al., 2012; Stitt and Zeeman, 2012). In support of the latter, the lack of major starch degradation suppression during photoperiod extension is compatible with evidence that it can occur even in the light under late-day/twilight regulation (Baslam et al., 2017; Fernandez et al., 2017; Ishihara et al., 2022; Stitt and Zeeman, 2012).

Although starch degradation may not be overly impaired under static continuous light, ongoing assimilation would continue its accumulation and can promote excessive net retained amounts, whereas the transition to dim nights in the Dynamic recipes offered an opportunity to unload net retained carbon (**Figure 7**; **Supplementary Figure 4**). Relative export exceeded 100% after the Dynamic recipes entered their dim phases, indicating that total export of labeled carbon outpaced assimilation and therefore required a contribution from previously assimilated labeled carbon during the light intensity step-down (Geiger and Fondy, 1979). Because Dynamic 1 and Dynamic 2 accumulated different retained labeled pools prior to their respective transitions, relative export magnitudes cannot be interpreted as a direct quantitative comparison of “efficiency” between Dynamic recipes. Also, because nighttime labeling began midway through the photoperiod, remobilization estimates based on labeled pools likely underrepresent contributions from non-labeled reserves formed earlier in the day or carried over from prior day cycles. Steady-state labeling would have to occur over several days continuously to truly estimate net carbon balance. Nevertheless, the consistent emergence of values >100% specifically after entry into dim phases provides a robust, semi-quantitative indicator that remobilization contributes measurably to export when concurrent assimilation is reduced. Accordingly, the retained labeled pool accumulated prior to each recipe’s transition strongly predicted the estimated remobilization component across Dynamic 1 and Dynamic 2 (**Supplementary Figure 5**). This relationship supports a pool-buffering interpretation in which dim transitions reveal remobilization demand relative to reduced assimilation, while the absolute contribution of remobilization remains constrained by the size of the retained pool present at the transition.

Finally, the illuminated post-label interval under Constant and dim-light phases of Dynamic treatments provides an important constraint for mechanistic interpretation. The negligible recovery of ¹^4^CO_2_ in CO_2_ traps during the post-label interval indicates that net loss of labeled CO_2_ from the labeled leaf was small (data not shown), consistent with strong reassimilation of internally released labeled CO_2_ under light (Busch et al., 2013). Thus, photorespiration is best framed here as influencing export coupling primarily through energetic/Pi constraints and partitioning control during the labeled feed, consistent with TPU/Pi frameworks (McClain and Sharkey, 2019; Yang et al., 2016). Also, it constrains net loss of label as CO_2_ during the post-label interval but does not preclude export of labeled carbon as organic products, including photorespiratory amino acids (e.g., glycine/serine) that can contribute to end-product synthesis and are transported in the phloem (Madore and Grodzinski, 1984; Sharkey, 2024; Tegeder and Hammes, 2018).

These results motivate future work: (i) chloroplast/cytosolic Pi and hexose-phosphate profiling across Day 1 to Day 4 Constant to test the Pi/TPU hypothesis; (ii) O_2_ manipulation (low vs. ambient O_2_) with concurrent steady-state ¹^4^CO_2_ export to quantify photorespiration-linked shifts in export coupling; (iii) starch-to-maltose/glucose dynamics across the post-label interval to distinguish reserve flux routing; (iv) transporter abundance/localization (SlSUT1/SlSUT4; tonoplast SUC4-type) to bound contributions of loading versus compartment buffering; and (v) SPS activation state (phosphorylation) and sucrose-phosphate/hexose-phosphate pools across the feed and post-label intervals to test whether reduced relative export reflects end-product commitment/TPU-Pi feedback rather than loading limitation.

## 5 Concluding thoughts regarding tomato/CEA carbon-balance modeling

Lighting optimization in CEA increasingly targets when photons are delivered (photoperiod extension and dynamic spectra/PPFD blocks) as well as how many photons are delivered (DLI), creating a need for crop models that resolve sub-daily carbon allocation and transport responses under rapidly changing light environments (Kuijpers et al., 2019). Classic tomato crop models such as TOMSIM represent biomass partitioning as a function of organ sink strengths and have been widely used to simulate dry matter distribution among vegetative organs and fruit trusses (Heuvelink, 1996). TOMGRO was explicitly developed for coupling to greenhouse environment models and emphasized that short time steps (minutes to hours) are needed to address control decisions within the day, while still updating growth state variables at longer time scales (Dayan et al., 1993). These model lineages provide a strong foundation for CEA applications, but they typically treat non-structural carbohydrate (NSC) storage as a simplified overflow or as a largely implicit buffer, which can be limiting when photoperiod extension and dynamic lighting intentionally impose strong within-day shifts in carbon input (Heuvelink, 1996; Zepeda et al., 2023).

A growing synthesis argues that carbon storage and remobilization behave as active buffers that support growth when carbon supply and sink demand are temporally asynchronous and, therefore, should be represented explicitly in plant and crop simulation models rather than as passive excess accumulation (Zepeda et al., 2023). In parallel, there is increasing recognition that phloem transport capacity and source–sink transport processes can constrain realized photosynthesis and reserve allocation, creating a “transport–assimilation capacity difference” that changes with environment and developmental context (Ainsworth and Bush, 2011; Leonardos et al., 2003; Vincent et al., 2025). This transport-capacity perspective is particularly relevant to extended photoperiods because carbon assimilation can be increased without proportional increases in export/transport or sink utilization, a situation expected to elevate leaf carbohydrate pools and alter feedback regulation of photosynthesis and growth (Ainsworth and Bush, 2011; Paul and Foyer, 2001; Zepeda et al., 2023). Greenhouse tomato work using source–sink analysis similarly emphasizes that the benefit of supplementary lighting depends on the balance between assimilate production and the plant’s capacity to utilize assimilates, motivating explicit quantification of source–sink limitation under specific lighting strategies (Li et al., 2019).

Within this modeling context, combined gas exchange and steady-state ¹^4^CO_2_ labeling provides unusually direct constraints on carbon-balance terms that are otherwise difficult to parameterize at sub-daily resolution. Steady-state labeling at isotopic equilibrium yields concurrent estimates of assimilation and export, enabling direct estimation of relative export and its diel evolution under defined lighting blocks (Geiger and Fondy, 1979; Grodzinski et al., 1998; Lanoue et al., 2018). The same framework yields a retained-pool state variable (newly assimilated labeled carbon not immediately exported) and, via the unlabeled chase, a reserve-supported export efficiency term describing how previous photoassimilates contribute to export during the post-label interval (Fondy and Geiger, 1982; Stitt and Zeeman, 2012). The present study illustrates why these quantities are informative for CEA-oriented models: relative export shifted during early continuous-light exposure, producing increased retention even when NCER was only modestly affected, and retained pool size strongly predicted export supported by previously assimilated pools in all photoperiod extension lighting conditions (excluding long-term photoperiodic injured treatment) (**Supplementary Figure 3**). Notably, concurrent export during the feed and chase-derived reserve-supported export behaved semi-independently (e.g., reduced relative export on Constant Day 4 without detectable suppression of proportional reserve-supported export), indicating that models should allow these processes to vary independently rather than assuming a fixed coupling between assimilation and export across diel states, at least for including extended-photoperiod effects into the models. Because TOMGRO-type models were designed to support greenhouse control applications with fast loops that respond to changing PPFD, these empirically derived export/retention/reserve-export parameters provide a principled way to implement (and validate) a “buffer plus export” module within existing source–sink frameworks (Dayan et al., 1993; Kuijpers et al., 2019).

A compact, implementation-oriented interpretation is that models including extended photoperiods should retain sink strength and transport feedback terms (Paul and Foyer, 2001) while adding at least two dynamic features that become critical when lighting is time-structured: (i) an explicit, active NSC (retained) pool in source leaves (or canopy) that can accumulate and be drawn down, and (ii) a potentially time-varying assimilation–concurrent export coupling term (relative export) that allows concurrent export to decouple from assimilation and generate transient retention (Ainsworth and Bush, 2011; Vincent et al., 2025; Zepeda et al., 2023). The results of this study are the first to indicate that the combination of steady-state labeling and an examination of ^14^C chase periods can be modified to identify these features because such techniques separate (a) concurrent export during assimilation from (b) export supported by previously assimilated reserves, on time scales relevant to dynamic LED recipes. Finally, because phloem export can include organic products beyond sugars (e.g., amino acids), tracer-derived relative export should be interpreted as whole-leaf carbon export unless phloem composition is measured (Tegeder and Hammes, 2018). As dynamic lighting becomes more common in CEA, integrating these measured state variables into tomato models should improve predictive performance under photoperiod extension by allowing the model to represent not only changes in photosynthetic capacity but also changes in allocation, buffering, and transport efficiency that determine carbon delivery to sinks (Paul and Foyer, 2001; Zepeda et al., 2023).

## Data Availability

The original contributions presented in the study are included in the article/**Supplementary Material**. Further inquiries can be directed to the corresponding author.
