# Supplementary material for: Steady-state 14CO2 tracing reveals early disruption of assimilation–export coupling in tomato under static continuous light but not under dynamic 24-h LED schedules
Source: Front Plant Sci. 2026 Jun 9;17:1781439. doi: 10.3389/fpls.2026.1781439 (PMC13289111; doi:10.3389/fpls.2026.1781439)
Supplement: Supplementary file 1 [file DataSheet1.docx]

# Supplementary Material

## Leaf gas exchange and ^14^C Export Measurements - extended

Leaf gas exchange and C export rates were obtained using an open-flow gas analysis and ^14^CO_2_ labeling system similar to that described previously (Lanoue et al., 2018, Leonardos et al., 1996, Grodzinski et al., 1998, Leonardos and Grodzinski, 2000, 2001, 2003, 2014). A personal computer (690 Precision, Dell) using a custom designed combination of data acquisition and control hardware (National Instruments Corporation, Austin, TX, USA) and software (LabView 2009, National Instruments Corporation) was used to control, monitor devises and log data. Control boards allowed for the measurement of analogue signals from sensors, and outputted digital and analogue signals to devices that controlled the environment of the gas flowing through the leaf chambers and over the leaves of measurement.

During experiments, plants were held in an illuminated whole-plant growth cabinet (GS20 BDAF LT big foot, Biochambers, Winnipeg, Canada) under constantly controlled irradiance, temperature, CO_2_ and humidity levels. Individual leaves on these plants were placed in specialized leaf chambers before the start of the photoperiod (08:00) and their gas exchange was monitored for 23.5h. The 1^st^ lateral leaflet of the 3^rd^ or 4^th^ true leaf was used as it was mature, fully expanded, and covered the entire leaf chamber/GM window. At this relatively large transplant age, ‘Money Maker’ had small floral development.

The system included four leaf chambers and a reference chamber. The middle portion of a leaf was enclosed in a brass leaf chamber which had been chrome plated to reduce problems associated with water exchange. The leaf chamber was consisted of a top part (16 cm^2^ exposing area through a glass window) and a bottom part in which was mounted a Geiger-Müller (GM) detector (model 7231, LND Inc., Oceanside, NY, USA). Both upper and lower sections of the leaf chamber were designed as water circulating jackets for leaf temperature control. Temperature of the water circulating through the leaf chamber was controlled by a water bath (RTE10 Neslab, Thermo Scientific). Leaf and air temperatures inside the leaf chamber were measured with two thermocouples (Type T model 5TC-TT-T-30-36 0.010 Dia./Ga., Omega Engineering Inc., Stanford, CT, USA) inside the leaf chamber. One thermocouple was placed in contact with the lower leaf surface (leaf temperature) and the other was under the leaf but not in contact with it (air temperature). Light (photosynthetic photon flux density (PPFD) 400-700 nm and photon flux density (PFD) 380-780 nm) was provided by LED fixtures as described in the lighting section above and was measured with a spectrometer (model LI-180, LI-COR Inc., Lincoln, NE, USA) positioned at the surface level of the leaf.

Compressed air was used to provide air for all leaf chambers. To avoid variations in the CO_2_ concentration in this compressed air during the day and over seasons, all of the air was passed through an FT-IR purge gas generator (model 75-52, Parker Balston, Analytical Gas Systems, Havenhill, MA, USA) to remove all CO_2_ and H_2_O from the air. The desired CO_2_ concentration (44 Pa) was obtained by mixing the CO_2_-free air from the purge gas generator with pure CO_2_ (Linde, Guelph, ON, Canada) using two mass-flow controllers (MFC) (Smart-Trak, 100 Series, Sierra Instruments, Inc., Monterey, CA, USA). The total volume of gas from the two MFC's (2 l min^-1^) entered a mixing/buffer container. Humidity (dew point) in the gas was controlled by passing the gas stream through a gas bubbler placed in a temperature controlled water bath (RTE17 Neslab, Thermo Scientific, Waltham, MA, USA). To avoid water condensation within the stainless steel tubing, all the gas lines were traced with a heating cable and maintained at temperatures well above the dew point. A ^14^CO_2_ injection and mixing loop was used during labeling experiments (see below). The main gas line was then split into a reference line and four sample lines one for each of the leaf chambers. The flow rate in each sample lines was maintained constant (0.4 l min^-1^) by a MFC (Smart-Track, 100 Series, Sierra Instruments Inc.). The flow rate in the reference line was controlled by a variable rate flow meter (Model N112-02, Cole-Parmer Instrument Co., Niles, IL, USA). The reference flow meter was also used to control the air pressure in the gas stream before the four sample MFC's. This air pressure was set at 2 psi, the minimum needed for the operation of the sample MFC’s, and was monitored by an inline pressure sensor (Cole-Parmer Instrument Co.). Each sample line passed through a leaf chamber. An electronic flow meter (Smart-Trak, Sierra Instruments, Inc.) precisely measured the flow rate in each line after the leaf chambers. The flow rate from this electronic flow meter was compared to the flow from the MFC before the leaf chamber and thus, used to detect potential leaks from each leaf chamber. A manifold of solenoid valves (Model 8320G222/3 Red-Hat, Asco, Florham Park, NJ, USA) was used to direct the gas of each line thought a CO_2_/H_2_O infrared gas analyzer (IRGA) (model 7000, LI-COR), one line at a time. The CO_2_ and H_2_O concentrations entering each leaf chamber (Reference line) and those exiting each leaf chamber (Sample line) were measured with the IRGA, thus, monitoring the gas exchange of each leaf. Using the recorded reference and sample CO_2_ and H_2_O concentrations, gas flow rate, leaf and air temperatures, and the leaf area enclosed in the leaf chamber, the rates of photosynthesis/respiration (net C exchange rate (NCER)), transpiration (E) and stomatal conductance to CO_2_ (g) were calculated.

Measurements of ^14^C-export were made during an initial 16-h steady-state ^14^CO_2_ labeling and a following 8-h chase period. Due to limitations of the ^14^CO_2_ injection system (minimum syringe injection speed), the maximum time of steady-state labeling was 16 h. After the leaves were inserted in the leaf chambers in the morning and the lights were turned on, ^14^CO_2_ was supplied at steady-state rate for 16 h (the entire photoperiod of the Control). Then the supply of ^14^CO_2_ was turned off and ^14^C was chased during the dark (Control) the “subjective night” period of extended light (Constant) or of low light (Dynamic 1 and 2). However, an additional set of experiments was also carried out with ^14^CO_2_ labeling starting in the afternoon (13:00) for 16 h to provide estimates of steady-state labeling ^14^C-export for the Constant and Dynamic1and 2 treatments during their “subjective night” periods.

^14^CO_2_ was generated by reacting Ba^14^CO_3_ (ViTrax, Inc., Placentia, CA, USA) with 30% HCl (Fisher Scientific, Toronto, ON, Canada) in a large airtight syringe (500 mL, Model S0500, Hamilton Company, Reno, NV, U.S.A). During steady-state ^14^CO_2_ labeling, labeled air taken with a 60 ml syringe (Becton, Dickinson and Company, Franklin Lakes, NJ, USA) was injected into the gas stream by using a precision syringe pump (PHD 2000 Infusion, Harvard Apparatus, Holliston, MA, USA). The specific activity of ^14^CO_2_ in the gas steam was monitored by a GM detector in the reference chamber in line after the ^14^CO_2_ mixing loop (see above). A standard curve (GM counts vs. specific activity) was obtained by trapping a volume (5 ml) of the inlet gas in ethanolamine/ethylene glycol monomethyl ether (1.0 ml; 1:2 v:v) (Fisher Scientific, Toronto, ON, Canada). Samples of the inlet gas were taken from a ^14^C-sampling port at which point the temperature of the gas was measured by a thermocouple (Type T model 5TC-TT-T-30-36 0.010 Dia./Ga., Omega Engineering Inc.). During each 16-h labeling run the specific activity of the ^14^CO_2_ in the supplied gas stream remained constant. However, this specific activity varied among experiments from 50-60 Bq μmol^-1^ C depending on the strength of ^14^CO_2_ generated for each set of experiments.

The GM detector positioned under the leaf surface was used to monitor the radioactivity accumulated/retained in the leaf during the steady-state labeling period in a manner similar to that described by Geiger and Fondy (1979). The GMs were controlled by a custom made power supply (model 045-001, Lou Champagne Systems Inc.) and the output through a rate counter (model NI-PCI-6602, National Instruments) was recorded continuously during the feed. At the end of the 24-h experiment each leaf was taken out of the chamber and the total radioactivity recovered in the fed portion of the leaf being over the GM detector was determined destructively by liquid scintillation counting. This final total retained counts and the final recorded GM counts were used to calculate the GM efficiency and correct the GM recorded counts and provide the rate of ^14^C retention during the entire 24-h experiment.

As described above, the NCER provided by the IRGA was used to measure the rate of ^14^C-assimilation/gain throughout the experiment. The rate of ^14^C-retention was also measured non-destructively by the GM detector under the leaf. ^14^C-export rates at any time during the steady-state labeling 16-h period was calculated as the difference between assimilation and retention rate. Relative ^14^C-export (% export) was calculated as the ratio of export to assimilation times 100. The rate of ^14^C-export during the 8-h chase period following the steady-state labeling was also obtained. The rate of ^14^C-retention (negative) from the source leaf during the chase was also continuously monitored by the GM detector. Additionally, the respiratory release of ^14^CO_2_ was determined during the chase by trapping of the outlet gas in 40 ml of ethanolamine/ethylene glycol monomethyl ether (1:2 v:v) and by liquid scintillation counting (Leonardos *et al.*, 1996). ^14^C-export during the chase was then corrected for respiration losses.

## Steady-State ^14^C Export Background - extended

Radiotracer experiments using ¹⁴CO₂ have long been used to follow the fate of newly fixed carbon, beginning with classic short-pulse labeling studies that helped map the carbon fixation pathway (Calvin & Benson, 1948; Calvin, 1961). In a pulse–chase design, leaves receive a brief ¹⁴CO₂ pulse followed by a chase in unlabeled CO₂, and the time course of label redistribution among soluble metabolites, starch, and transport products is used to infer pathway turnover and fluxes (Baslam et al., 2017; Kruger et al., 1983). Pulse–chase approaches are well suited to resolving kinetics of specific pools, but they do not inherently provide a continuous, intact-leaf accounting of export versus retention, because the specific activity of carbon in exportable pools changes throughout the pulse and chase and export is typically inferred indirectly from pool labeling rather than measured as a leaf-scale flux (Geiger & Fondy, 1979). Methodological constraints (e.g., coarse operational definitions of “starch label,” contamination of enzymatic digests from non-starch compounds, and limited ability to detect rapid cycling) can also influence pulse–chase inferences about turnover in illuminated leaves (Baslam et al., 2017; Kruger et al., 1983).

To move from endpoint partitioning toward direct export flux estimation, early translocation work in sugar beet established sucrose as the dominant translocated product under steady photosynthesis (Geiger & Swanson, 1965). Building on this foundation, Geiger and Fondy developed a nondestructive method for continuous export estimation from an intact source leaf by coupling open-flow gas exchange with continuous monitoring of leaf radioactivity using a Geiger–Müller detector positioned adjacent to the leaf (Geiger & Fondy, 1979). In this framework, leaf ¹⁴C retention is monitored continuously (as the change in leaf radioactivity over time, corrected for counting efficiency), and export is calculated as the difference between the rate of ¹⁴C fixation and the rate of ¹⁴C retention, allowing treatment-driven changes in export to be resolved at ~10–20 min time resolution when the labeled carbon distribution and counting geometry are sufficiently stable (Geiger & Fondy, 1979).

Modern steady-state ¹⁴CO₂ feeding extends this logic by supplying a constant ¹⁴CO₂ source until the specific activity of the major transport sugars approaches isotopic equilibrium while infrared gas analysis provides concurrent measurements of CO₂ assimilation and transpiration (Geiger & Fondy, 1979; Grodzinski et al., 1998). Under these conditions, concurrent (immediate) export can be estimated during ongoing photosynthesis with reduced ambiguity relative to non-steady-state labeling (Grodzinski et al., 1998). When labeling spans the photoperiod and is followed by a chase in unlabeled CO₂, the diel fate of newly fixed carbon can be partitioned into (i) export during the light, (ii) export after labeling ends (remobilization-supported export), (iii) respiratory loss, and (iv) retained label, which aligns with questions about daytime versus nighttime sources of exported carbon (Grodzinski et al., 1998; Lanoue et al., 2018).

Because newly fixed ¹⁴C does not instantaneously mix through the pools supplying export, data acquired early in the feed period are not used for steady-state export calculations. Across diverse species, isotopic equilibrium of transport sugars often required ~60–90 min after initiating steady-state labeling, and Grodzinski et al. used data after ≥90 min (e.g., 90–120 min) to estimate concurrent export under stable photosynthesis (Grodzinski et al., 1998). In tomato steady-state labeling studies, a comparable equilibration window has been applied prior to interpreting concurrent export and relative export trajectories (Lanoue et al., 2018). After step changes in PPFD during an ongoing feed, the time required for the retention/export signals to stabilize is expected to be shorter than at the start of labeling because major transport pools are already partially labeled; in the present study, an operational stabilization period of ~20 min after PPFD steps was used before analyzing post-step export dynamics (present study). This stabilization time reflects convergence of the leaf-scale retention/export signals rather than a verified isotopic steady state of all intermediary pools, which would require destructive pool-specific sampling around step transitions (Grodzinski et al., 1998; Lanoue et al., 2018).

## ^14^C Export calculations - extended

At each logging interval, net CO₂ exchange rate (NCER; µmol CO₂ m⁻² s⁻¹) was obtained from open-flow infrared gas analysis, while leaf radioactivity was monitored continuously with a Geiger–Müller detector positioned at a fixed geometry relative to the leaf. Following the steady-state ¹⁴CO₂ export framework (Geiger & Fondy, 1979; Grodzinski et al., 1998), the retention rate of labeled C was calculated, using the GM counts during the feed $R\left( t \right)$(Bq), a correction factor for the measurement geometry *f* (GM efficiency; Bq Bq^-1^, determined by the final GM counts and the counts recovered after extraction of the fed leaf area over the GM at the end of feed), the CO_2_ specific activity in the air stream, $SA_{CO_{2}}$(Bq µmol⁻¹ CO₂), and leaf area in the leaf chamber, LA (m-2) (Geiger & Fondy, 1979; Lanoue et al., 2018). This retention rate was defined as the time derivative of leaf labeled C content:

$$\mathrm{Retention}_{C}\left( t \right)=\frac{dR\left( t \right)}{dt} \times f \times\frac{1}{SA_{CO_{2}}} \times\frac{1}{LA}$$

During the ¹⁴CO₂ feed, the C fixation rate was computed from NCER.:

$$\mathrm{Fixation}_{C}(t)=\mathrm{NCER}(t)$$

The export rate of labeled C from the source leaf was then calculated as:

$$\mathrm{Export}_{C}(t)=\mathrm{NCER}(t)-\mathrm{Retention}_{C}(t)$$

expressed in µmol CO₂ m⁻² s⁻¹, and determined once the exportable pools approached isotopic equilibrium, usually after 60-90 min of steady-state labeling in most species (Grodzinski et al., 1998; Leonardos and Grodzinski, 2014).

The relative export fraction was calculated as:

$$\mathrm{Relative}\mathrm{Export}_{C}(t)=\frac{\mathrm{Export}_{C}(t)}{\mathrm{NCER}(t)}\times100\%$$

and was evaluated only during intervals with positive NCER and after the isotopic-equilibrium period.

Time-integrated totals over defined windows were computed by numerical integration of the instantaneous rates:

$$\mathrm{Total}\text{ }\mathrm{fixed}\text{ }C=\int\mathrm{NCER}(t)\text{ }dt,\mathrm{Total}\text{ }\mathrm{exported}\text{ }C=\int\mathrm{Export}_{C}(t)\text{ }dt,$$

with the retained pool formed during the feed computed as:

$$\mathrm{Retained}\text{ }= Total\text{ }\mathrm{fixed}\text{ }\text{- }\mathrm{Total}\text{ }\mathrm{exported}\text{ }C,$$

consistent with steady-state ¹⁴CO₂ accounting used to partition fixation into concurrent export versus retention (Grodzinski et al., 1998; Leonardos and Grodzinski, 2014; Lanoue et al., 2018).

During the unlabeled chase (no ¹⁴CO₂ in the air stream), there is no fixation of supplied label and changes in $R(t)$ reflect net loss of previously fixed label from the leaf. We operationally defined chase export / remobilization as the carbon-equivalent loss of label from the source leaf over a chase window. However, because there was a negative NCER (respiration) for the Control treatment which was under dark during the chase, the chase export estimates were corrected for the respiration label losses determined by the radioactivity was recovered in respiration traps. For the Constant and both Dynamic 1 and 2 the NCER were positive during the chase periods and no radioactivity was found in their respiration traps.

For step changes in PPFD during an ongoing feed, an operational stabilization interval was applied before calculating post-step export metrics, reflecting the time required for the retention/export signals to settle after the perturbation (Geiger & Fondy, 1979; Lanoue et al., 2018).

## References - extended

Calvin, M. (1962). The path of carbon in photosynthesis. *Science, 135*(3507), 879–889. https://doi.org/10.1126/science.135.3507.879.

Calvin, M., & Benson, A. A. (1948). The path of carbon in photosynthesis. *Science, 107*(2784), 476–480. https://doi.org/10.1126/science.107.2784.476.

Geiger, D. R., & Swanson, C. A. (1965). Sucrose translocation in the sugar beet. *Plant Physiology, 40*(4), 685–690. https://doi.org/10.1104/pp.40.4.685.


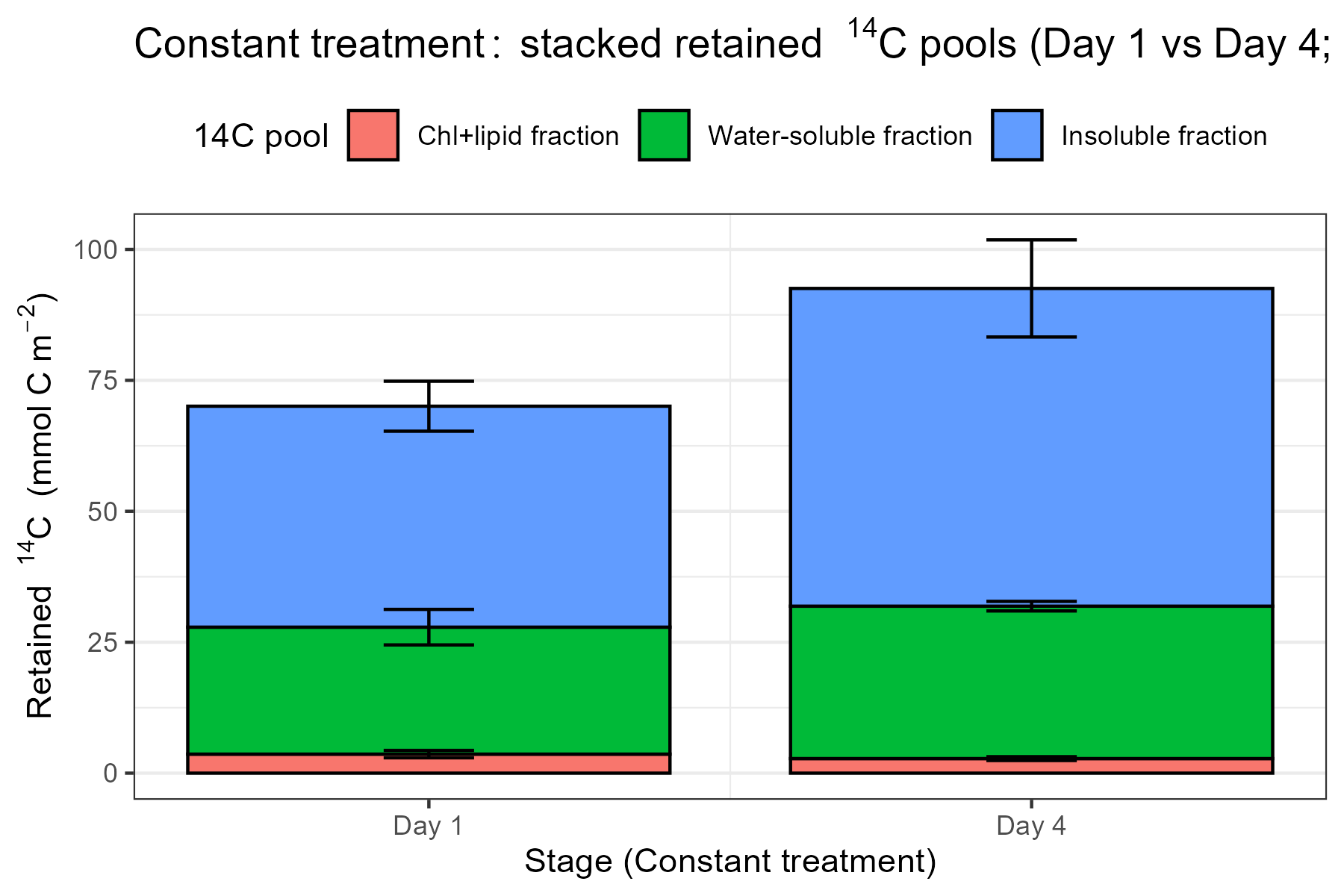


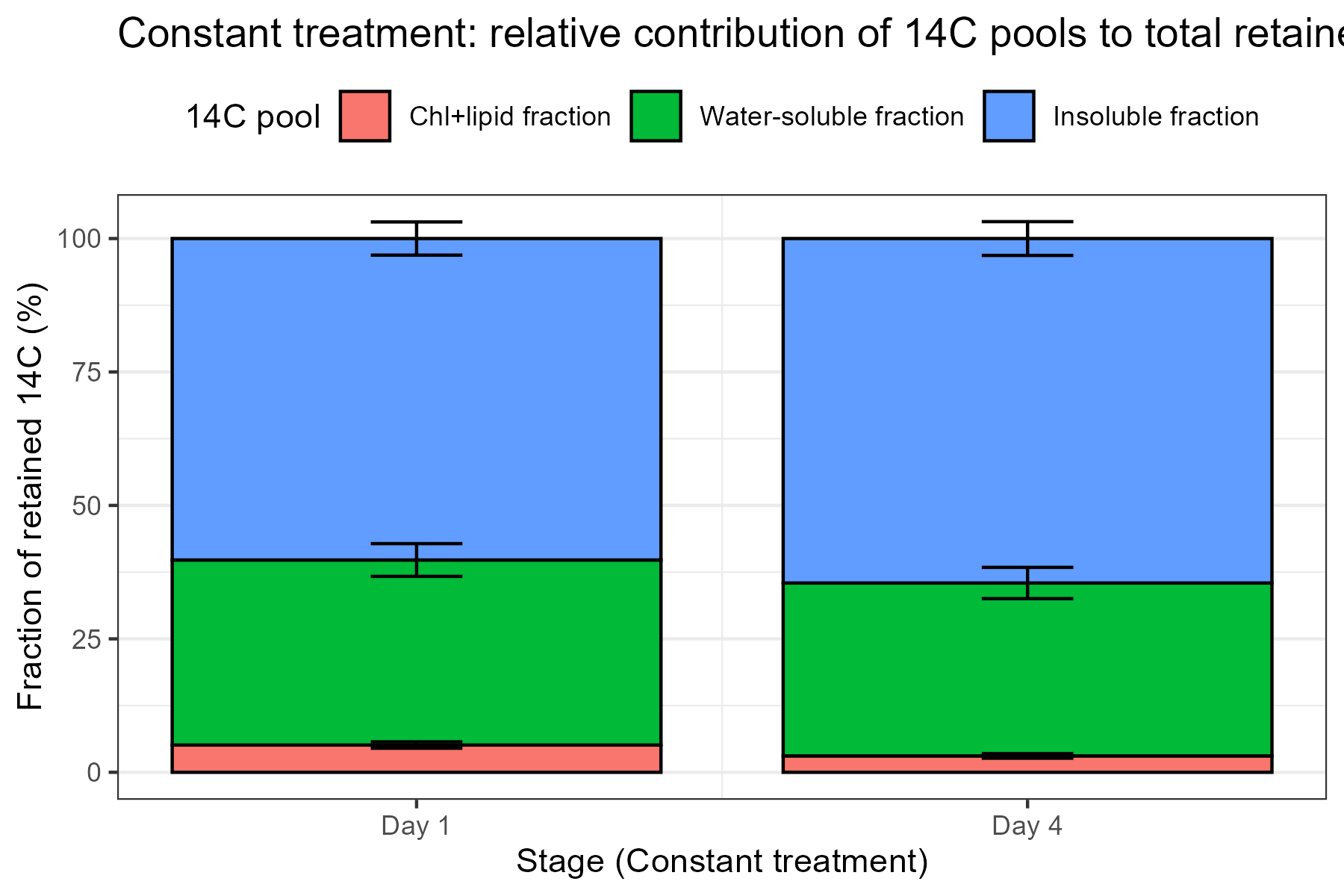


**Figure S1.** Partitioning of retained ^14^C among biochemical pools at the end of the 24-h for the Daytime feed runs. Bars show the mean retained ^14^C per leaf for Constant on Day 1 and Day 4. Within each bar, coloured segments represent the chloroform fraction (bottom), water-soluble fraction (middle), and insoluble fraction (top) quantified from scintillation counting of fractionated leaf tissue. Values shown as mean ± SE.

**Table S1.** Short-term continuous-light stress response (Days 1–4**).** Phase-shifted dynamics and treatment differences in peak window activity under constant light. Data include average export rate (mmol h⁻¹), total export (mmol), and export %. Statistical significance denoted by * (p < 0.05), ** (p < 0.01), ***(p<0.001).

| **Variable** | **Phase shift (Day4−Day1) [h]** | **Peak-window mean (Day4−Day1)** | **Week3 − Day1 (acrophase mean difference)** |
| --- | --- | --- | --- |
| NCER | 9.503 ± 0.847** | -0.765 ± 0.408 | -4.465 ± 0.506** |
| Exp | 5.927 ± 0.580** | -0.693 ± 0.473 | -3.447 ± 0.468** |
| Export % | 3.357 ± 0.780* | -7.299 ± 3.031† | 6.617 ± 1.547* |
| E | 8.600 ± 0.632*** | -0.154 ± 0.039* | 0.023 ± 0.086 |
| WUE | 7.952 ± 0.593*** | 0.214 ± 0.261 | -6.627 ± 0.199*** |
| GCO2 | 8.915 ± 0.731** | -24.596 ± 4.759* | -9.211 ± 8.993 |
| Ci | 8.565 ± 0.790** | -43.083 ± 5.537** | 96.028 ± 6.053*** |


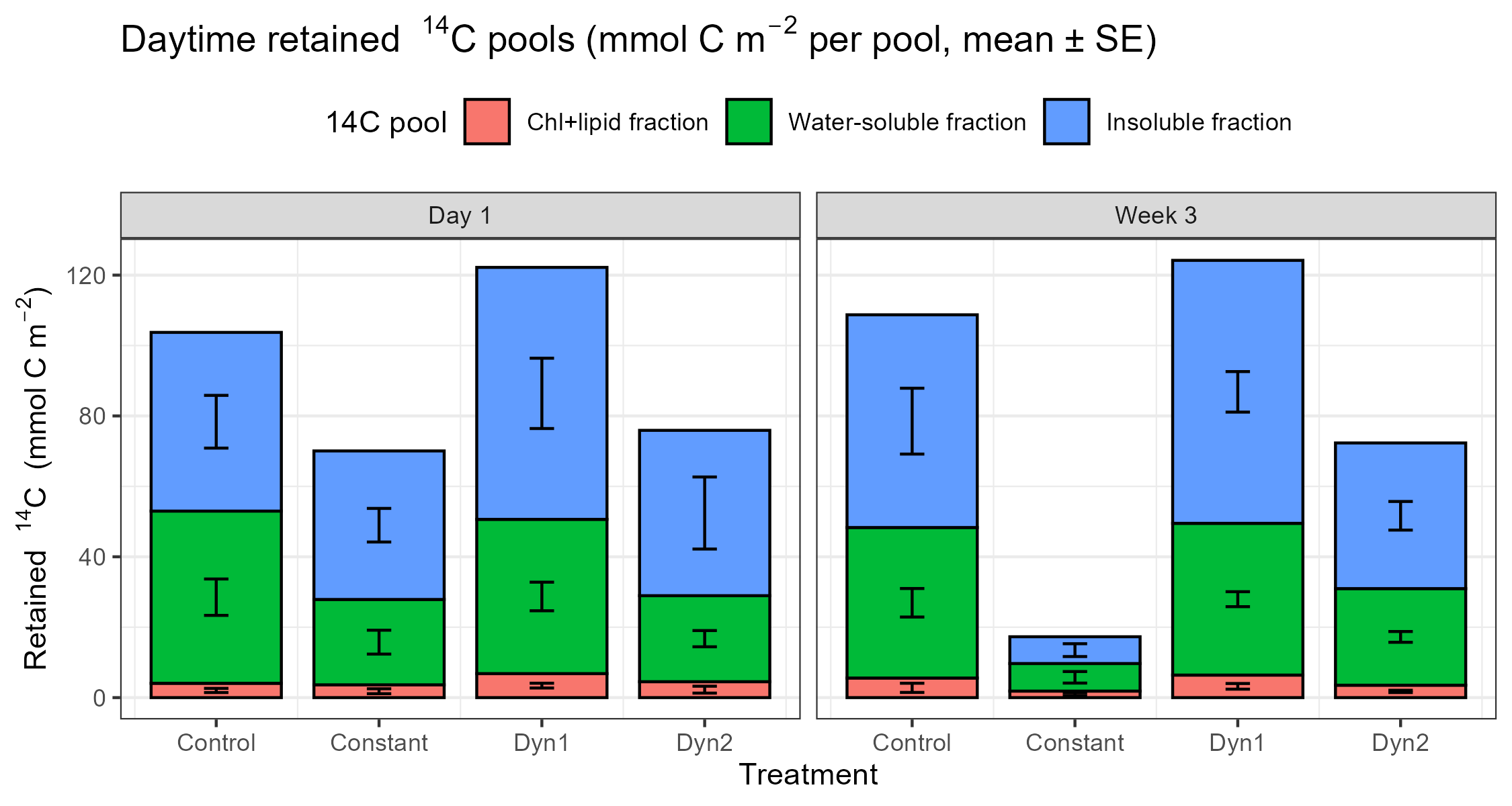

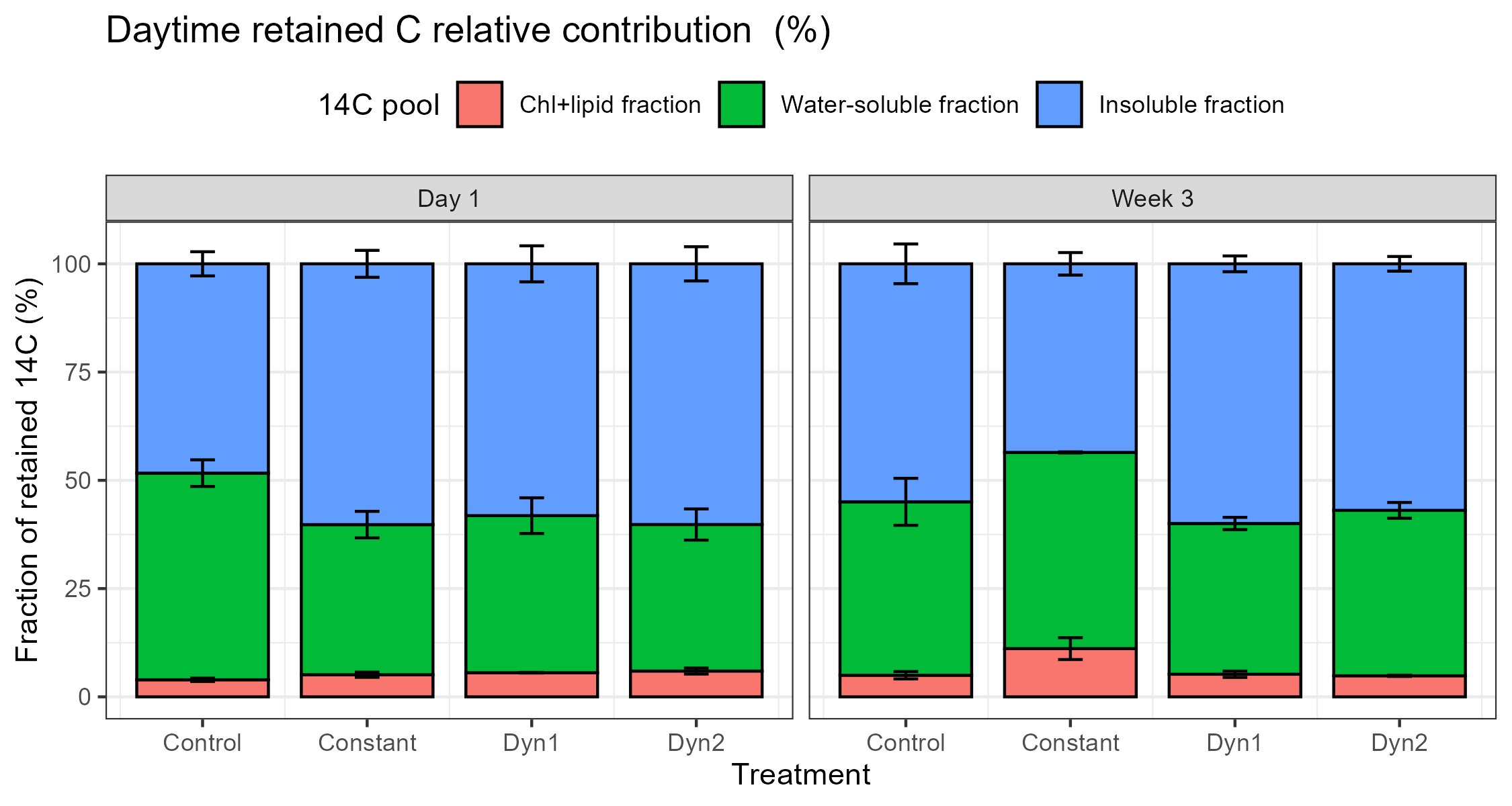

**Figure S2.** Partitioning of retained ^14^C among biochemical pools at the end of the 24-h Daytime feed runs. Bars show the mean retained ^14^C per leaf for each lighting treatment (Control, Constant, Dynamic 1, Dynamic 2) on Day 1 and after 3 weeks of acclimation (Week 3). Within each bar, coloured segments represent the chloroform fraction (bottom), water-soluble fraction (middle), and insoluble fraction (top) quantified from liquid scintillation counting of fractionated leaf tissue. Values shown as mean ± SE.

## ****Table S2.** Whole-day (0–24 h) physiological responses across light treatments and acclimation. LS-adjusted means ± SE (n = 4 plants per treatment × day)** for total fixed carbon, mean net carbon exchange rate (NCER), transpiration (E), stomatal conductance (gCO₂), and whole-day water-use efficiency (WUE), integrated or averaged over the full diel cycle (0–24 h). For total fixed C, a two-way ANOVA revealed significant effects of **Treatment** (F₃,₂₄ = 23.68, p = 2.37 × 10⁻⁷), **Day** (F₁,₂₄ = 25.44, p = 3.72 × 10⁻⁵), and a strong **Treatment × Day interaction** (F₃,₂₄ = 20.43, p = 8.53 × 10⁻⁷), indicating treatment-specific acclimation responses. Simple-effects analyses showed no treatment differences on Day 1 (F₃,₁₂ = 0.88, p = 0.48), but strong treatment effects after three weeks (F₃,₁₂ = 60.32, p = 1.65 × 10⁻⁷), driven primarily by a collapse in Constant light. When Constant was excluded a priori, treatment differences at Week 3 remained significant (F₂,₉ = 4.43, p = 0.0457). For transpiration, two-way ANOVA showed a significant main effect of **Day** (F₁,₂₄ = 6.72, p = 0.016), but no effect of **Treatment** (p = 0.63) and no interaction (p = 0.87). Stomatal conductance showed no significant effects of Treatment (p = 0.62), Day (p = 0.99), or their interaction (p = 0.45). In contrast, whole-day WUE exhibited significant effects of **Treatment** (F₃,₂₄ = 32.24, p = 1.39 × 10⁻⁸), **Day** (F₁,₂₄ = 102.87, p = 3.73 × 10⁻¹⁰), and a strong **Treatment × Day interaction** (F₃,₂₄ = 30.57, p = 2.29 × 10⁻⁸). Mean NCER is shown descriptively; inferential statistics for carbon gain are based on total fixed C.

| Treat | Total fixed C (mmol C m⁻² d⁻¹) | NCER (µmol m⁻² s⁻¹)* | Transpiration E (mmol m⁻² d⁻¹) | | Stomatal conductance gCO₂ (mol m⁻² s⁻¹) | | WUE (µmol CO₂ mmol⁻¹ H₂O) | |
| --- | --- | --- | --- | --- | --- | --- | --- | --- |
| Day 1 | | | |  | |  | |  |
| Control | 526.4 ± 17.6 | 9.4 ± 0.6 | 4.87 ± 0.22 | | 0.31 ± 0.02 | | 6.89 ± 0.07 | |
| Constant | 559.7 ± 21.3 | 6.9 ± 0.3 | 4.92 ± 0.19 | | 0.33 ± 0.01 | | 10.45 ± 0.27 | |
| Dyn1 | 578.8 ± 26.7 | 9.3 ± 0.7 | 4.78 ± 0.25 | | 0.32 ± 0.02 | | 9.69 ± 0.51 | |
| Dyn2 | 593.4 ± 14.8 | 8.2 ± 0.3 | 4.83 ± 0.18 | | 0.30 ± 0.01 | | 11.06 ± 0.80 | |
| Week 3 | | | |  | |  | |  |
| Control | 526.9 ± 13.9 | 8.7 ± 0.5 | 5.21 ± 0.28 | | 0.30 ± 0.02 | | 6.67 ± 0.24 | |
| Constant | 201.6 ± 18.9 | 2.5 ± 0.5 | 5.09 ± 0.31 | | 0.29 ± 0.03 | | 3.30 ± 0.35 | |
| Dyn1 | 560.9 ± 15.5 | 9.1 ± 0.4 | 5.14 ± 0.21 | | 0.31 ± 0.02 | | 8.20 ± 0.11 | |
| Dyn2 | 580.4 ± 8.0 | 8.1 ± 0.4 | 5.19 ± 0.24 | | 0.32 ± 0.02 | | 8.78 ± 0.16 | |

**Table S3.** Nighttime chase export dynamics and remobilization efficiency. Export rate (µmol C m⁻² s⁻¹) and export total (mmol C m⁻²) were calculated for two chase windows following the end of daytime labeling at 16–18 h and 18–24 h. Retained label at 16 h (Ret₁₆; mmol C m⁻²) was calculated as the integral of (NCER − Exp) from 1.5–16 h (post–isotopic equilibrium), and mobilization efficiency was computed as (window export total / Ret₁₆) × 100%. Values are least-squares means ± SE (n = 4 replicates per treatment × acclimation). Acclimation effects on export rate and export total were tested using one-way ANOVAs within each treatment (factor: Acclim, Day 1 vs Week 3); the only significant acclimation effect occurred under Constant lighting during 18–24 h, where export rate and export total decreased from Day 1 to Week 3^1^, and these Week 3 cells are denoted by an asterisk in the table. Mobilization efficiency was analyzed by two-way ANOVA with factors Treatment and Acclimation. Significant Treatment × Acclimation interactions were detected for both windows (16–18 h^2^ and 18–24 h^3^. Accordingly, simple effects were tested using one-way ANOVAs of mobilization efficiency among treatments within each acclimation^4^, followed by Tukey-adjusted pairwise comparisons. Different letters within a given day and window indicate significant treatment differences (Tukey HSD, α = 0.05). Acclimation effects on mobilization efficiency within each treatment were tested by one-way ANOVA (Day 1 vs Week 3)^5^.

| Treatment | Export rate (µmol C m⁻² s⁻¹) | | Export total (mmol C m⁻²) | | | Remobilization efficiency (Export_window_ / Retained_1.5-16_ * 100%) | |  |
| --- | --- | --- | --- | --- | --- | --- | --- | --- |
| Day 1  16-18 h | |  | |  | | |  |  |
| Control | 2.94 ± 0.22 | | 21.14 ± 1.60 | | | 15.20 ± 1.40 [a] | |  |
| Constant | 1.29 ± 0.25 | | 9.31 ± 1.82 | | | 12.81 ± 1.59 [a] | |  |
| Dyn1 | 2.62 ± 0.23 | | 18.89 ± 1.68 | | | 12.99 ± 1.18 [a] | |  |
| Dyn2 | 1.21 ± 0.25 | | 8.72 ± 1.79 | | | 10.89 ± 1.62 [a] | |  |
| Day 1 18-24 h | |  | |  | | |  |  |
| Control | 1.12 ± 0.07 | | 24.21 ± 1.51 | | | 17.39 ± 1.40 a | |  |
| Constant | 0.55 ± 0.07 | | 11.81 ± 1.52 | | | 16.63 ± 1.37 a | |  |
| Dyn1 | 1.51 ± 0.14 | | 32.60 ± 2.99 | | | 22.27 ± 1.44 a | |  |
| Dyn2 | 0.64 ± 0.09 | | 13.88 ± 1.98 | | | 17.46 ± 1.20 a | |  |
| Week 3 16-18 h | |  | |  | | |  |  |
| Control | 2.67 ± 0.12 | | 19.20 ± 0.84 | | 13.92 ± 1.00 b | | | |
| Constant | 0.66 ± 0.11 | | 4.79 ± 0.76 | | 28.58 ± 2.82 a* | | | |
| Dyn1 | 2.71 ± 0.31 | | 19.54 ± 2.25 | | 13.01 ± 0.85 b | | | |
| Dyn2 | 1.75 ± 0.23 | | 12.61 ± 1.64 | | 15.96 ± 2.16 b | | | |
| Week 3 18-24 h | |  | |  | | |  |  |
| Control | 1.19 ± 0.14 | | 25.66 ± 2.97 | | 18.27 ± 1.39 b | | | |
| Constant | 0.32 ± 0.01* | | 6.98 ± 0.32* | | 44.97 ± 7.84 a* | | | |
| Dyn1 | 1.42 ± 0.18 | | 30.63 ± 3.87 | | 20.24 ± 1.11 b | | | |
| Dyn2 | 0.85 ± 0.08 | | 18.44 ± 1.70 | | 23.19 ± 1.52 b* | | | |

## ^1^(F(1,6)=9.615, p=0.0211). ^2^Treatment F(3,24)=8.955, p=0.00037; Acclim F(1,24)=16.807, p=0.00041; interaction F(3,24)=10.513, p=0.00013. ^3^ Treatment F(3,24)=6.962, p=0.00156; Acclim F(1,24)=14.602, p=0.00083; interaction F(3,24)=10.238, p=0.00016). ^4^(16–18 h Day 1: F(3,12)=1.463, p=0.274; 16–18 h Week 3: F(3,12)=14.641, p=0.000259; 18–24 h Day 1: F(3,12)=3.641, p=0.0447; 18–24 h Week 3: F(3,12)=9.143, p=0.00200). ^5^16–18 h—Constant F(1,6)=23.676, p=0.00281; Control F(1,6)=0.548, p=0.487; Dyn1 F(1,6)=0.00031, p=0.986; Dyn2 F(1,6)=3.540, p=0.1089; 18–24 h imputed—Constant F(1,6)=12.685, p=0.0119; Control F(1,6)=0.1976, p=0.672; Dyn1 F(1,6)=1.240, p=0.308; Dyn2 F(1,6)=8.802, p=0.0251.


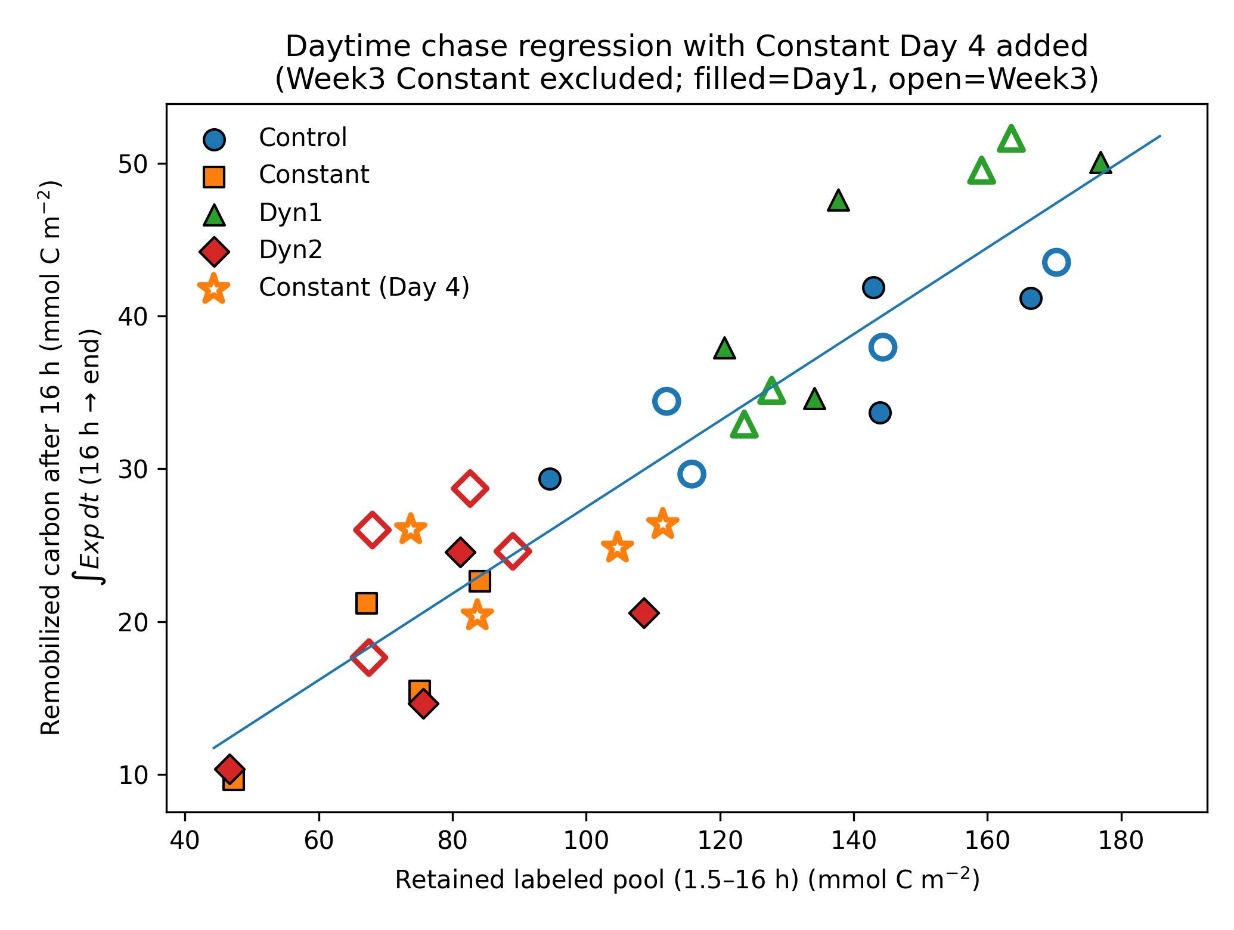

**Figure S3.** Daytime-labeled chase regression revealed that across Day 1 and Week 3 (excluding Week 3 Constant, and including Constant Day 4), post-16 h remobilized export scaled positively with the retained labeled pool (1.5-16 h) (slope = 0.286 ± 0.023, t(26)=12.28, p=2.49×10⁻¹², R²=0.853; intercept n.s.).


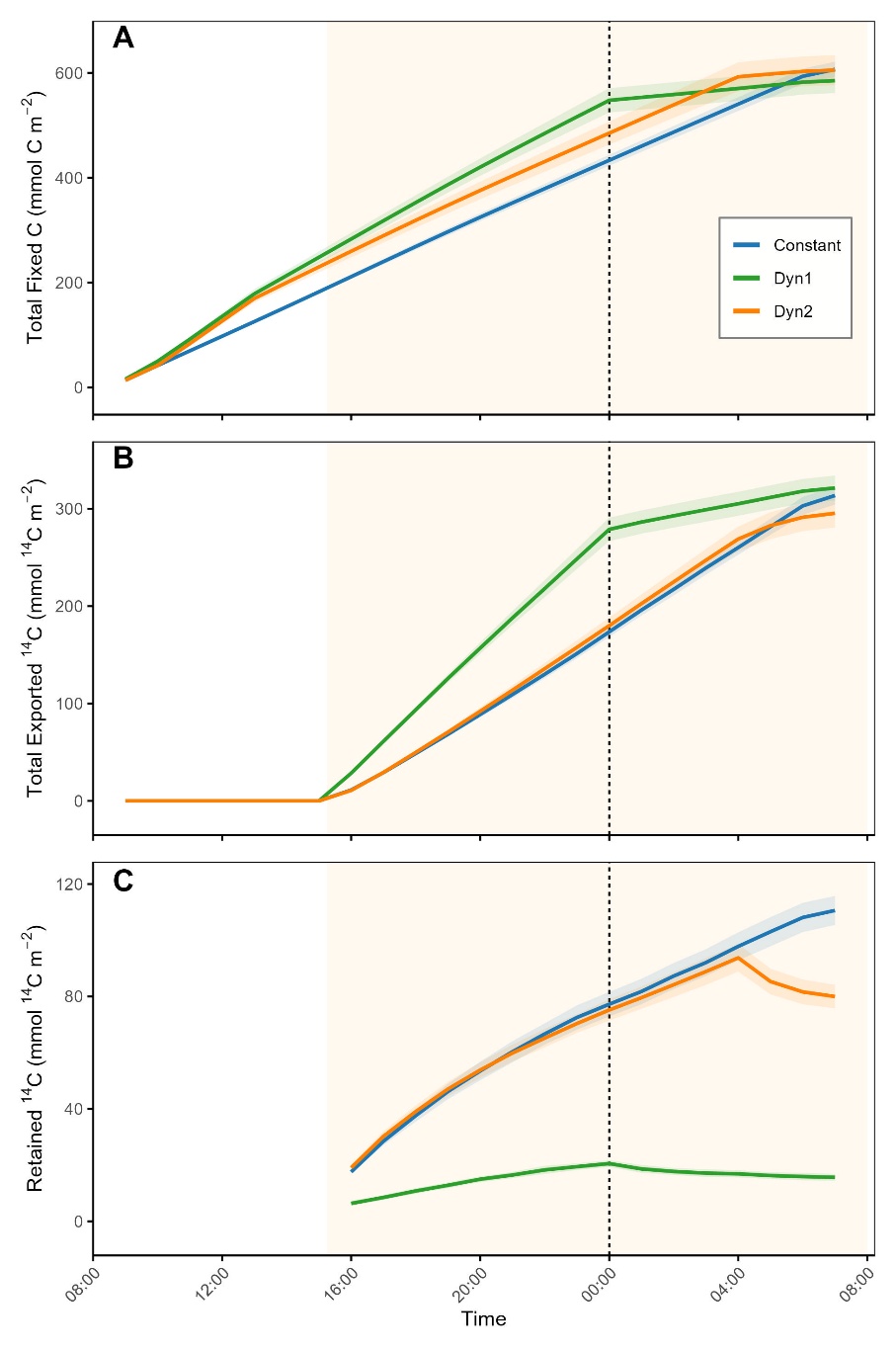

**Figure S4.** Nighttime labeled ^14^C feed showing the Total Fixed C (A; mmol C m⁻²), Total Export C (B; mmol C m⁻²), and Retained C in the source leaf (C; mmol C m⁻²) during the first day of treatments Constant (blue), Dynamic 1 (green), and Dynamic 2 (orange). ^14^CO2 was fed for 16 h starting mid-way through the photoperiod (~15:30) and continued throughout subjective nighttime (yellow shaded area). Tomato plants were previously entrained to 16 h photoperiods (noted as a dashed vertical line at 00:00). They were analyzed for photoperiod extension effects by comparing a 2 h window prior to expected photoperiod end (red background) and a 2 h window immediately after photoperiod extension (light blue background). There was no chase period in this experiment. Lines are means ± SE (n = 4).


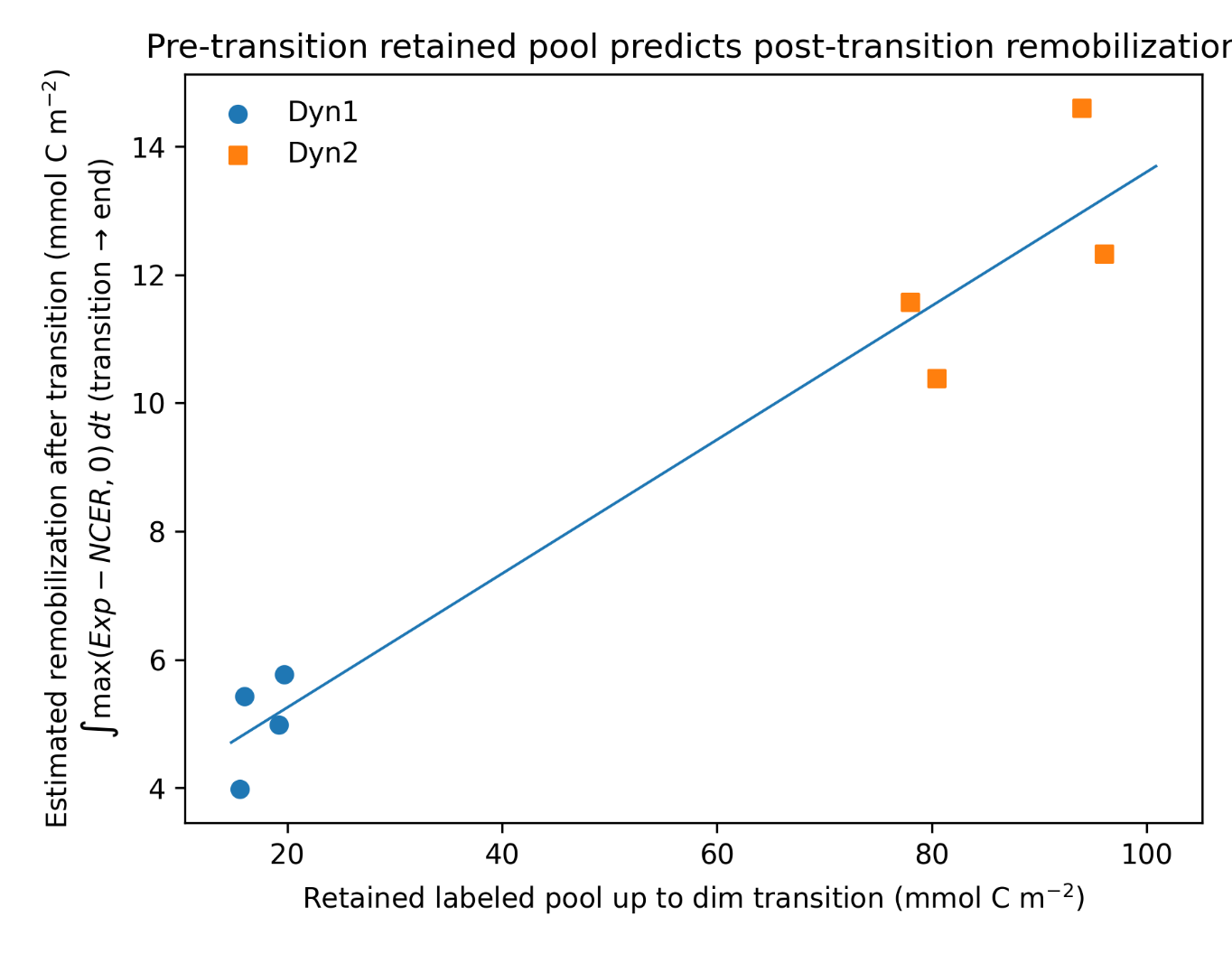

**Figure S5**. Pre-transition retained labeled pool predicts estimated post-transition remobilization in Dynamic treatments. Points show individual replicates for Dynamic 1 (circles) and Dynamic 2 (squares); line shows the pooled linear regression (n = 8). Estimated remobilization was calculated as $\int\max(\mathrm{Exp}-\mathrm{NCER},0)\text{ }dt$from the dim-light transition (16 h for Dyn1; 20 h for Dyn2) to the end of the run (24 h). Linear fit: slope = 0.1044, R^2^ = 0.946, p = 5.0 X 10^-5^. Note, because labeling began in the latter portion of the photoperiod, remobilized carbon will include unlabeled reserves formed earlier, so these regressions should only be qualitatively compared with daytime labeled chase regression coefficients (Fig. S4).


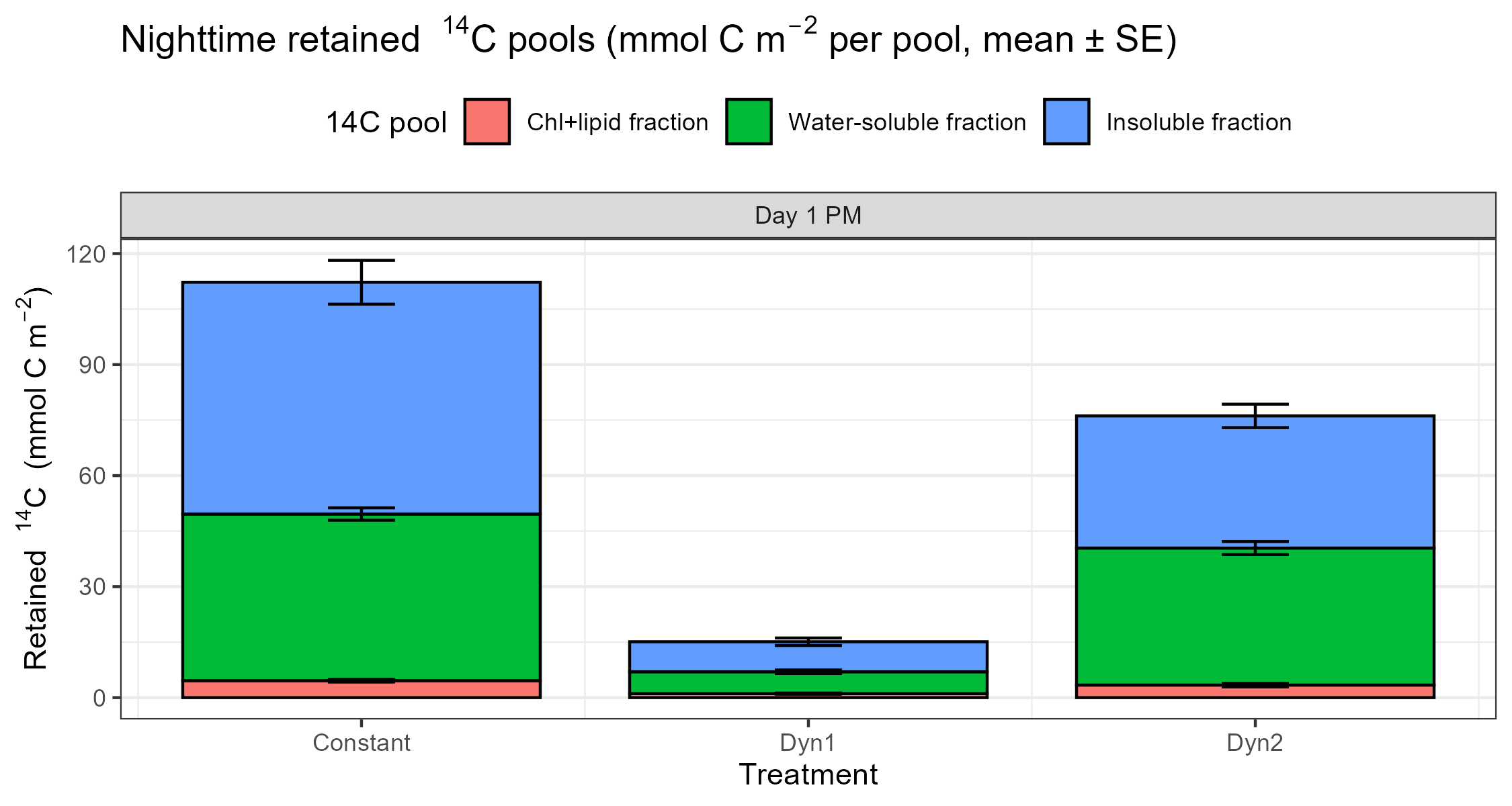


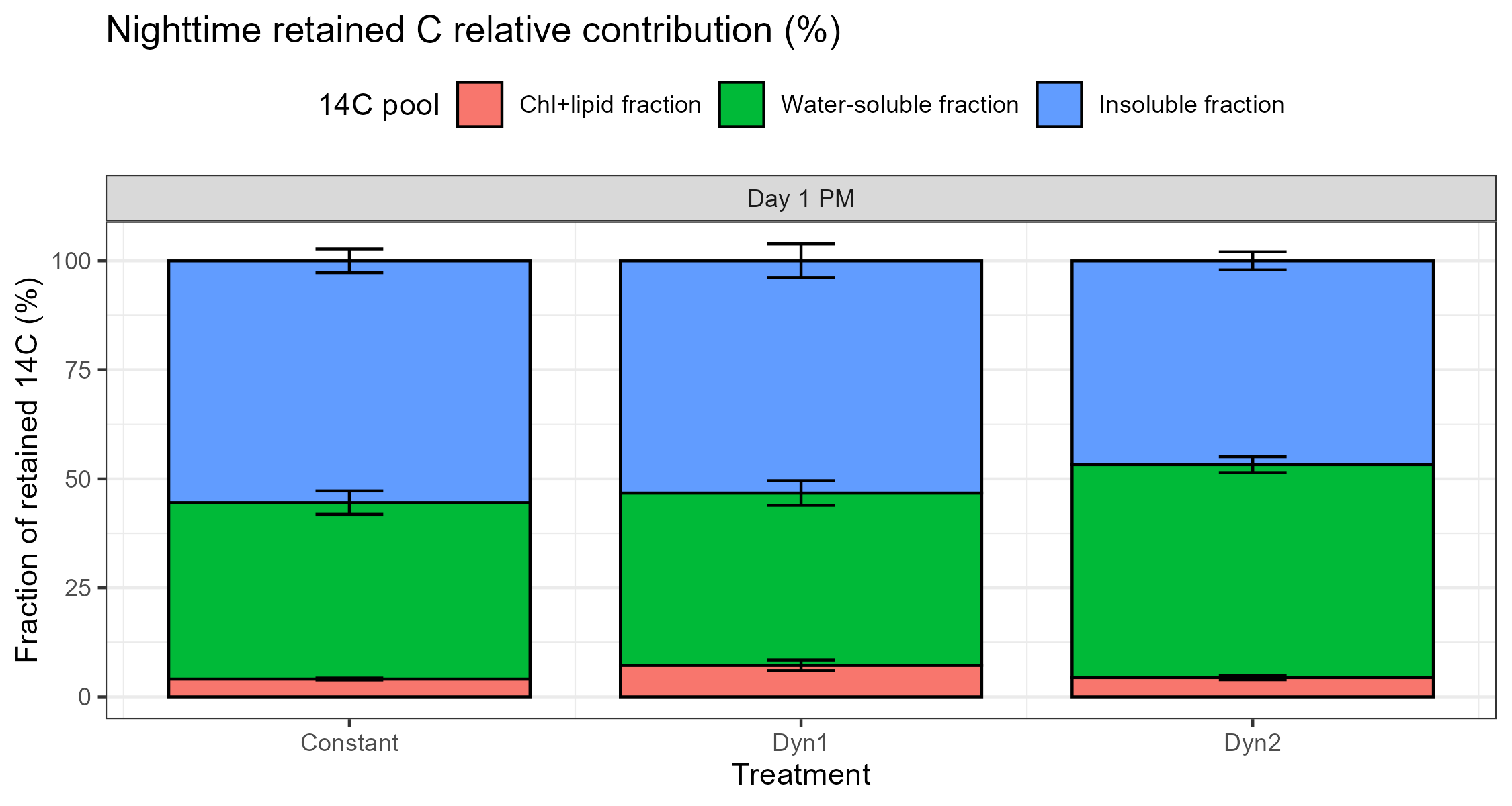

**Figure S6.** Partitioning of retained ^14^C among biochemical pools at the end of the 24-h for the Nighttime feed runs. Bars show the mean retained ^14^C per leaf for each lighting treatment (Constant, Dynamic 1, Dynamic 2) on Day 1. Within each bar, coloured segments represent the chloroform fraction (bottom), water-soluble fraction (middle), and insoluble fraction (top) quantified from scintillation counting of fractionated leaf tissue. Values shown as mean ± SE.


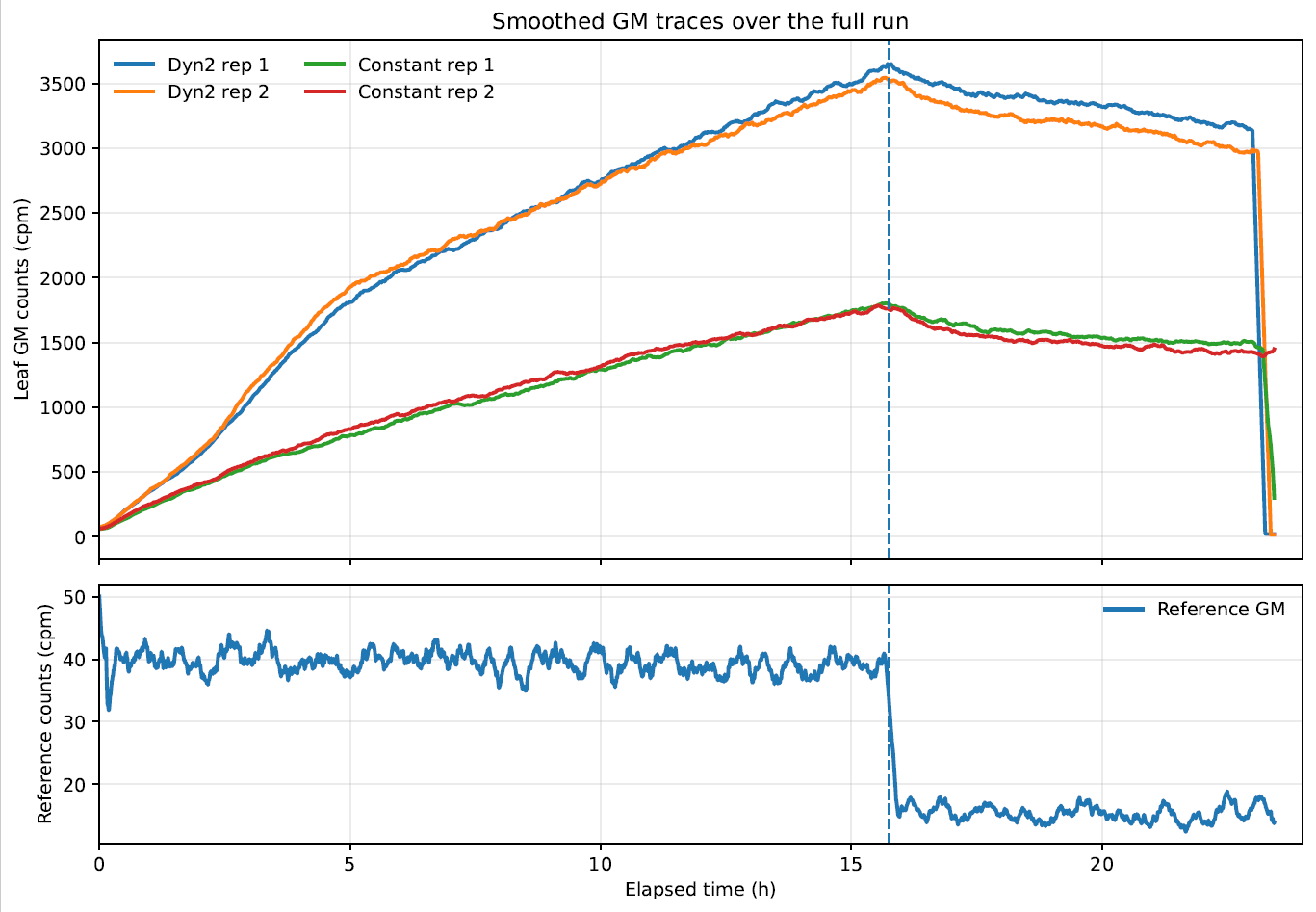

**Figure S7**. An example of a Geiger-Muller counter trace from 4 leaves during a run and a reference leaf chamber, both used for calculating retained carbon.
